# Harvesting Candidate Genes Responsible for Serious Adverse Drug Reactions from a Chemical-Protein Interactome

**DOI:** 10.1371/journal.pcbi.1000441

**Published:** 2009-07-24

**Authors:** Lun Yang, Jian Chen, Lin He

**Affiliations:** 1 Bio-X Center, Key Laboratory for the Genetics of Developmental and Neuropsychiatric Disorders (Ministry of Education), Shanghai Jiao Tong University, Shanghai, China; 2 Institute of Biomedical Sciences, Fudan University, Shanghai, China; 3 Institute for Nutritional Sciences, Shanghai Institutes of Biological Sciences, Chinese Academy of Sciences, Shanghai, China; Stanford University, United States of America

## Abstract

Identifying genetic factors responsible for serious adverse drug reaction (SADR) is of critical importance to personalized medicine. However, genome-wide association studies are hampered due to the lack of case-control samples, and the selection of candidate genes is limited by the lack of understanding of the underlying mechanisms of SADRs. We hypothesize that drugs causing the same type of SADR might share a common mechanism by targeting unexpectedly the same SADR-mediating protein. Hence we propose an approach of identifying the common SADR-targets through constructing and mining an *in silico* chemical-protein interactome (CPI), a matrix of binding strengths among 162 drug molecules known to cause at least one type of SADR and 845 proteins. Drugs sharing the same SADR outcome were also found to possess similarities in their CPI profiles towards this 845 protein set. This methodology identified the candidate gene of sulfonamide-induced toxic epidermal necrolysis (TEN): all nine sulfonamides that cause TEN were found to bind strongly to MHC I (Cw*4), whereas none of the 17 control drugs that do not cause TEN were found to bind to it. Through an insight into the CPI, we found the Y116S substitution of MHC I (B*5703) enhances the unexpected binding of abacavir to its antigen presentation groove, which explains why B*5701, not B*5703, is the risk allele of abacavir-induced hypersensitivity. In conclusion, SADR targets and the patient-specific off-targets could be identified through a systematic investigation of the CPI, generating important hypotheses for prospective experimental validation of the candidate genes.

## Author Summary

Why do tragedies caused by Vioxx or Avandia only happen to certain individuals? The unexpected bindings among drugs and human proteins might play important roles in such serious adverse drug reactions (SADRs). To mine these unexpected chemical-protein interactions, 162 drug molecules known to cause SADRs are ‘hybridized’ onto 845 proteins to construct a chemical-protein interaction matrix, from which two aspects of the information, the binding strength and the binding conformation, are disclosed. Followed by the data-mining strategies, the unexpected bindings that mediate SADRs are identified. For example, abacavir is found to bind to the antigen presentation groove of MHC I molecule in patients carrying the B*5701 allele but not B*5703, which explains why HLA-B*5701, not B*5703, is the risk allele of abacavir hypersensitivity. This research could explain to the public that SADR happens when some of the innocent proteins are attacked by drugs unexpectedly, and variances in certain people's genome make their proteins more sensitive to the drug. By pre-therapy screening, the susceptible people could be protected. Furthermore, new drugs or modified drugs will be designed to avoid these patient-specific unintended bindings, in a step toward realizing personalized medicine.

## Introduction

Identifying genetic risk factors responsible for serious adverse drug reactions (SADRs) is one of the priorities in pharmacogenetics [1]. As it is not practical to perform genome-wide association study due to the lack of samples [2], candidate gene selection has been an important strategy. Challenges arise when the primary mechanisms for many SADRs are unclear. Consequently, the candidate genes selected are generally limited to those coding therapeutic targets [3], transporters or metabolic enzymes [4]. We named this strategy as the “known interaction driven selection”, for it is driven by the known interactions between drugs and proteins. However, the drug-protein interactions at this level cannot explain why an SADR is only induced by certain medications but never caused by other drugs. For example, Stevens-Johnson syndrome (SJS) is often caused by diclofenac, didanosine and tenoxicam but never caused by propoxyphene. It is undisputed that direct interaction between a chemical and a protein, for example, noncovalent binding of a drug to the active center of an enzyme, is a fundamental step in drug effect. Hence, we hypothesized that drugs causing the same type of SADR might share a common mechanism by targeting unexpectedly on the same SADR-mediating protein. But questions still arise as why after strict assessment before the drugs came to the market, SADRs still and only happen to some certain individuals, especially the type B or idiosyncratic SADR [5]. Evidences showed that some rare polymorphisms within these SADR targets made them more sensitive to the drug. For example, oseltamivir (Tamiflu), an anti-flu drug, whose active form binds to the active site of human cytosolic sialidase. A rare polymorphism near the binding pocket may enhance this unexpected binding and might increase susceptibility to oseltamivir-induced neuropsychiatric disorders [6]. Another case concerns the A1555G mutation in mitochondrion DNA, which enhances the unwanted binding of aminoglycosides to human 12s rRNA, mediating the susceptibility of aminoglycosides-induced deafness [7]. Thus, the candidate gene selection of SADR genetics can be tackled by exploring the unexpected chemical-protein bindings. To harvest them at high throughput, we established the first chemical-protein interactome (CPI) in the form of the interaction strength among FDA-approved drugs and human proteins. Each of the drugs was reported to cause at least one of the four major SADRs including SJS/TEN, cholestasis, rhabdomyolysis and deafness. We designed a data-mining strategy against the CPI to explore whether the common SADR targets existed. In brief, if different drugs that share the common outcome of SADR “S” all interact with a particular protein “P”, whereas drugs that do not cause the “S” outcome do not interact with it, then the common target “P” can be considered as a mediator of “S” and prioritized for association studies and mechanism investigations.

## Results

### Reliability of the DOCK program based CPI in assessing the chemical-protein interaction

Several techniques such as BIACORE biosensors [8] and drug affinity pull-down [9] can be used to assess the chemical-protein interactions and to identify the unexpected chemical-protein bindings. However, in order to test the utilities of CPI in a low cost and high throughput manner, we intended to choose a mature technique. Dock programs [10] appeared to be a good option. The DOCK [11] has been under development and improvement for over 20 years and is widely used to evaluate the interaction strength between drug candidates and proteins targets. Particularly, it has previously been used to identify the unexpected binding. A classical case is haloperidol, an anti-psychiatric drug, which was found to bind unexpectedly to HIV protease and had become a template for developing anti-HIV drugs [12]. The discovery was made with DOCK, whose later version [13] was applied in our research reported here.

Since human knowledge of the four SADRs is limited, neither did we know any of the unintended drug-protein interactions in them nor did we not know how many protein targets should be enough to cover the mechanism space of them without bias. So we selected targets from literature and third-party targetable protein database [14]–[17] and then applied quality control steps as described in the methods. Considering our productivity of pockets preparation and the urgency of solving the SADR problem, a set of 845 proteins (Table S1) finally passed the quality control. Although the protein set was incompetent to cover the whole SADR targets, if some unexpected and valuable information could be mined from it, the methodology of CPI would enlighten the following research and thus lead to the construction of a large scale target set. We constructed a test CPI to test the feasibility of using DOCK in evaluating the interactions. In our protein set, there were 12 proteins which had been the therapeutic targets as they were listed in DrugBank [18]. We extracted the known direct interactions with the 12 proteins reported in DrugBank or the literature for a total of 46 drugs. We then ran the DOCK for 46×891 times, resulting in a docking score matrix with 46×891 elements. The matrix was then shrunk to 46×845 elements before we converted the matrix into the Z-score [19] matrix, where binding affinities for each drug across the 891 binding pockets were normalized to a mean of zero and standard deviation of one, since it has been reported that the normalization of the docking score matrix can improve the hit ratio [20]. Docking score distribution is dependent on sizes, charges and other characters of the drug. The normalization could address this inconsistency among drugs. Each drug-protein interaction was classified into one of the two categories, depending on whether (group 1) or not (group 2) the interaction was previously reported in DrugBank or in the literature. Statistical test of Z-score showed that the two groups belonged to different population (Table S2). The area under ROC curve was 0.74 (95% CI: 0.68–0.80, Figure S1), indicating that the Z-score was valuable in measuring true bindings. The 50^th^ percentile of Z-score in group 1 interactions was −1.240 while the 50^th^ percentile of group 2 was only −0.47. We thus set a Z-score threshold of −1.2 in order to distinguish the known bindings (group 1) from the unidentified bindings (group 2). Note that some of the unidentified bindings might exist *per se*, which would reduce the difference between two groups. However, this misclassification of unidentified interactions into known bindings did not affect the specificity of highlighting the true bindings from the unidentified ones. So we concluded that Z-score could tell whether a binding will occur at the interval of high absolute value.

Based on the reliability of the dock program and the data processing strategy that effectively separate known bindings from unidentified ones, we introduce the first release of CPI in the form of a Z-score matrix. The chemicals selected here were FDA-approved drugs, each of which was reported to cause at least one of the four major well-known SADRs mentioned above. The derivatives of these drugs, such as the known major metabolites and their known isomers were also included. In summary, the CPI consists of the binding affinity data between 162 chemicals and 891 binding pockets. The interaction strengths were converted into a Z-score matrix. We did not choose SADRs like hepatotoxicity, for it is a relatively big concept compared with cholestasis and can be induced by almost every drug. The four SADRs included in this research were not only the major SADRs usually reported in the FDA's Adverse Event Reporting System (AERS), but each of which was also appeared to be triggered by a particular set of medications. This type of SADR allowed us to identify “case” and “control” drugs from which clear differences in the pattern of binding to many proteins were observed from the CPI. In the following section, we used the SJS/TEN SADR as an example to illustrate the construction and utilities of the CPI.

### Identification of candidate genes of serious cutaneous reactions through mining the CPI

The SJS and TEN are two forms of the same life-threatening cutaneous reactions that cause rash, skin peeling, and sores on the mucous membranes triggered by particular types of medications [21] with primary mechanism unknown. No significant association was observed between the metabolite enzyme genes and the SJS/TEN [22], implying that “known interaction driven” genes might not be the fundamental element. We first selected drugs that were reported to be associated with this SADR in peer-reviewed publications. All of the drug-SJS/TEN relationships were confirmed in the FDA's AERS. In total, 32 drugs along with their 21 major derivatives served as the case group, whereas 17 drugs were verified to be unrelated to SJS/TEN in both publications and AERS and served as the control group, which did not contain the derivatives. To avoid biases in the following assessment, we also confirmed that they did not share the same chemical features.

**Figure 1:**
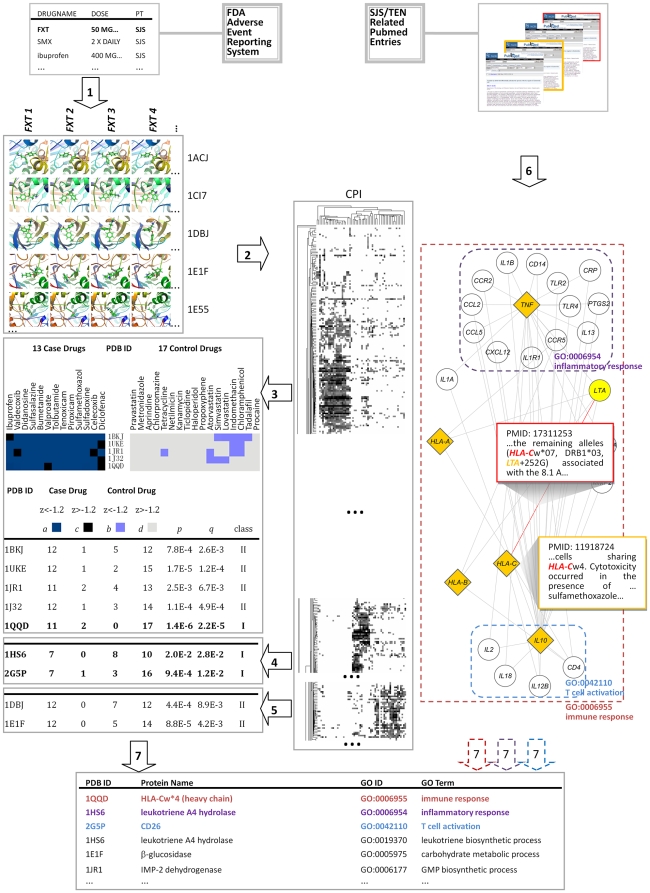
Strategy of identifying candidate genes for SADR through data-mining against the CPI and text-mining. **Step (1)** Drugs retrieved from adverse events reporting system of FDA were docked into the active sites of proteins. Shown here are the binding conformations of the four forms of fluoxetine (FXT1: the parent drug; FXT2: the major metabolite norfluoxetine; FXT3 and FXT4: nitrogen-atom positively charged of FXT1 and FXT2 respectively) to the bioactive sites of the five proteins. **Step (2)** Visualization of clustering results against the case matrix of binding affinity between 53 drugs (columns) and 845 proteins (rows). All case drugs were known to cause SJS/TEN. A Z-score greater or less than −1.2 were represented as white or black squares respectively. **Step (3)** A local CPI. Interactions of 13 case drugs in the sub-CPI1 and 17 control drugs with five proteins were shown here. For each of the proteins, the number of case drugs that interact with it with a Z-score<−1.2 (dark blue square) or with Z-score>−1.2 (black) were denoted as *a* and *c*, whereas the number of control drugs that interact with it with a Z-score<−1.2 (light blue) or with a Z-score>−1.2 (gray) were denoted as *b* and *d*, respectively. **Step (4) & (5)** Representative proteins highlighted from sub-CPI2 and sub-CPI3. The number of case or control drugs varied because of the missing values. **Step (6)** A local SJS/TEN-oriented GCCN. Yellow diamonds and white circles represent the core genes and extended genes respectively. One PubMed entry [26] (yellow bolded) referring to *HLA-C* mainly deals with TEN. Another entry [27] (red bolded) describes the relationship between *HLA-C* and *LTA* (red line). Genes that are annotated with GO term “inflammatory response” (in purple rectangle), “T cell activation” (blue) and in “immune response” (red) tend to be cited more specifically in this GCCN. **Step (7)** If a protein highlighted from the CPI share the GO terms enriched from GCCN, its symbol is presented in the corresponding color of the GO terms in step (6).

After docking all 70 molecules into all 891 binding pockets of our set of 845 proteins (step 1 of Figure 1), we obtained a Z-score matrix of the binding affinities. We then split it into the case matrix (53×845 relations) and the control matrix (17×845 relations). We performed a hierarchical clustering [23] on the resulting zero-floored Z-score matrix, and found that three sub-groups of case drugs clearly interacted selectively with three different sub-groups of proteins (step 2 Figure 1), implying that the three different sub-groups of case drugs might trigger the SJS/TEN through three different mechanisms. Then we divided the case matrix into three sub-CPIs, and performed a trimming procedure to exclude the redundant case chemicals if multiple forms of a drug were clustered into the same sub-CPI. To identify proteins preferentially interacting with the case drugs, we performed Fisher's exact tests followed by false discovery rate (FDR) corrections [24] for every sub-CPI in comparison to the control group. This non-parameter test only required binding information in a binomial pattern, which could be specifically measured by Z-score. In sub-CPI 1 (step 3 of Figure 1), *p* and *q* values [24] for each proteins were calculated. As a result, HLA-Cw*4 heavy chain (1QQD) together with other proteins were highlighted. In sub-CPI2 and sub-CPI3, leukotriene A4 hydrolase (1HS6), CD26 (2G5P) and Fab′ fragment of IgG (1DBJ) together with other proteins were identified (step 4, 5 of Figure 1).

### Identification of biological processes from the SJS/TEN-oriented gene co-citation network

We further enriched the biological process (BP) terms of Gene Ontology (GO) from the gene co-citation network (GCCN) [25]. We downloaded 18566 SJS/TEN-related PubMed entries, calculated the citation rates for each of the human genes in SJS/TEN related and nonrelated PubMed entries. Genes cited more specifically in the SJS/TEN topic were defined as core genes. Two genes were connected if they were co-cited in a PubMed entry (step 6 of Figure 1). To retrieve a more delicacy set of GO BP terms for SJS/TEN, we extended the core gene set through indexing their neighbors in GCCN. For example, *LTA* was one of the neighbors of the core gene *HLA-C* [26]. Though had not been investigated in SJS/TEN, *LTA* was co-cited with *HLA-C* [27], implying a putative functional linkage of this gene to SJS/TEN through *HLA-C*. Both the extended and the core genes were used in the enrichment analysis of BP terms [28]. As shown in step 6 of Figure 1, genes annotated with “immune response” (*p* = 8.32E-07), “inflammatory response” (*p* = 2.48E-18) and “T cell activation” (*p* = 1.02E-41) tended to occur more frequently in SJS/TEN-oriented GCCN than random selection at a significance level of 0.01.

### Highlighting the candidate genes for serious cutaneous reactions

**Figure 2:**
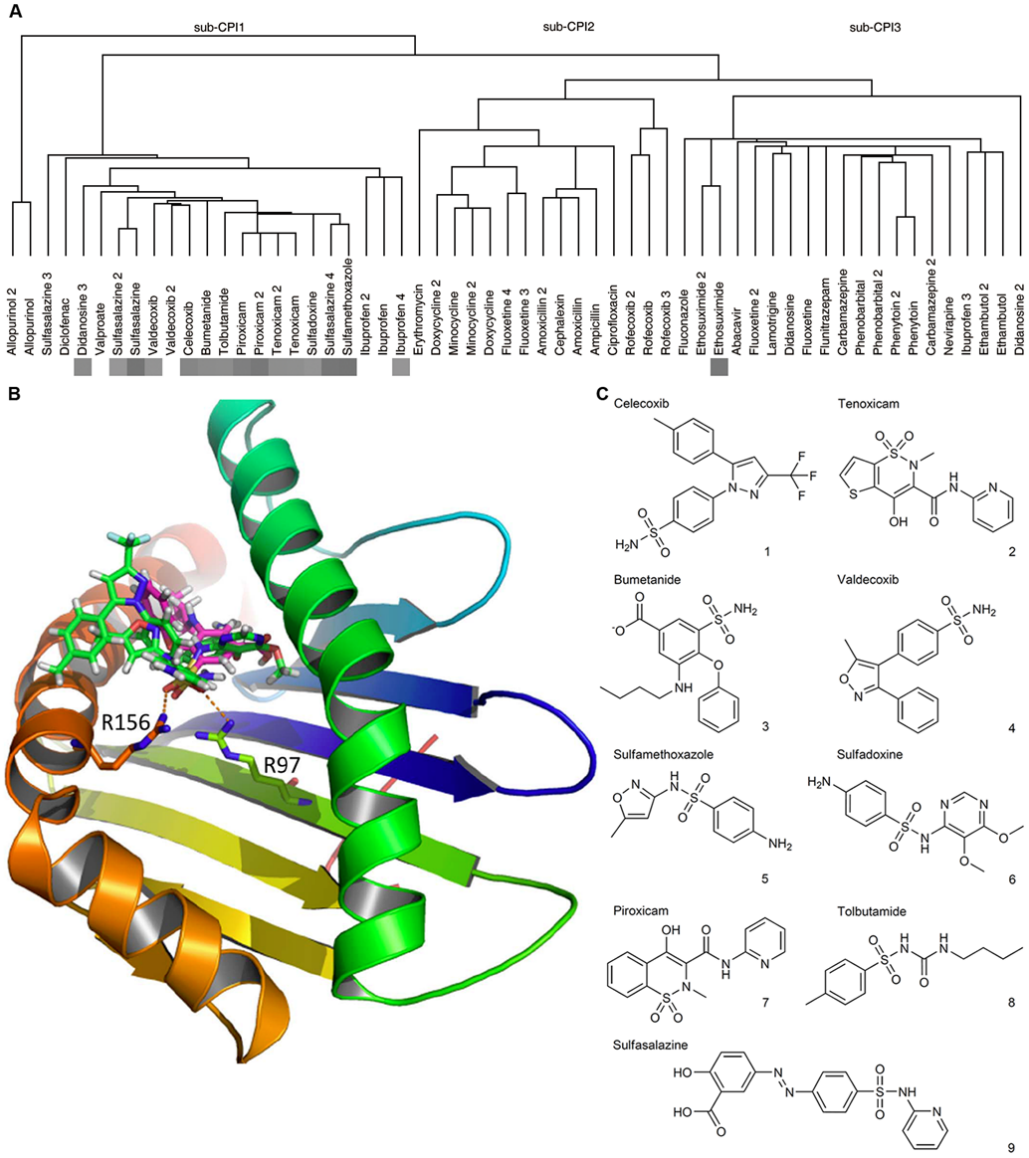
The interaction of sulfonamides to MHC I (Cw4). (*A*) Interaction strength among case drug molecules of SJS/TEN and MHC I (Cw*4). Drug names followed by the numbers represent the derivatives of this drug. In sub-CPI 1, 15 molecules interact strongly with MHC I (Cw*4). After trimming procedure, 13 case drugs including 9 sulfonamides (listed in C) were found binding to MHC I (Cw*4). (*B*) The lowest energy conformations of four sulfonamides' binding to the antigen presentation groove of MHC I (Cw*4) through hydrogen bonds between the oxygen atoms in sulfuryls to the nitrogen atoms in the two arginine residues (R97 and R156). Residues in the two α-helixes also contribute to the binding. The four sulfonamides shown here are bumetanide, celecoxib, sulfadoxine and sulfamethoxazole. See Figure S2 for the binding conformations of other sulfonamides. (*C*) The nine sulfonamides have different structures, but they all bind to the two stretched arginine residues through their sulfuryls as the ‘root’. Molecular structures of the 17 control drugs which do not tend to interact with MHC I (Cw*4) were shown in Figure S3.

Finally, the BP terms were assigned manually to proteins highlighted from sub-CPIs. Proteins were defined as the class I candidates if they shared the same BP terms enriched from GCCN. Otherwise they were defined as class II candidate. Class I candidates were involved in the known biological processes of SJS/TEN while class II proteins did not. However, it does not mean that class II candidates are less important since human knowledge on SJS/TEN is still limited. MHC I protein heavy chain Cw*4 (1QQD) showed the lowest *p* value in the candidate list. It is assigned as a class I candidate, as it was annotated with the BP terms “immune response” (GO:0006955) and “antigen processing” (GO:0019882), which were highlighted from the GCCN. When investigated the interaction strength among all case-control drugs and HLA-Cw*4 (Table S3), we found that 85% (11 of 13) case drugs including 9 sulfonamides bind strongly to it. Table S4 showed significant differences between case and control interactions with HLA-Cw*4 either in docking scores or in Z-scores. By visualizing the binding conformations at the lowest energy, we found that all sulfonamides tended to “root” at MHC I's antigen presentation groove through the hydrogen bond interaction of sulfuryls to the two arginine residues (Figure 2*B*). This identification of *HLA-C* (w*4) as the mediator of sulfamethoxazole (SMX)-induced TEN was validated by other studies, as it was confirmed that the immune response and the TEN will only be triggered by SMX in presence of MHC I (Cw*4) [26],[29].

Following the same data-mining and text-mining pipeline, we identified candidate genes from the other two sub-CPIs (Table 1). Two representative proteins highlighted were leukotriene A4 hydrolase (1HS6) and F_ab_ fragment of IgG (1DBJ). The former is the rate-limiting enzyme in formation of leukotriene, which transduces the signal of inflammation in skin reactions [30]. Case drugs tend to bind to its peptidase active center and might interfere the suicide regulation [31] of the enzyme itself when the enzyme is over-expressed. Case drugs in sub-CPI 3 tend to bind to the variable region of a certain IgG. We could not deduce the downstream events of this binding, but it has been known that the binding of antigens to the IgG induces the release of leukotriene, activate alexin system and the type III hypersensitivity.

**Table 1:** Candidate proteins mediating the four SADRs.

SADR	PDB	Protein Name	GO ID	GO Term	*p*	*q* a	*a*	*b*	*c*	*d*	sub-CPI	Class
SJS/TEN	1QQD	HLA-Cw*4(Heavy Chain)	GO:0019882	antigen processing and presentation	2.15E-05	2.98E-05	11	0	2	17	1	I
SJS/TEN	2G5P	CD26 (DPP4)	GO:0042110	T cell activation	9.39E-04	0.0124	7	3	1	16	2	I
SJS/TEN	1HS6	Leukotriene A4 hydrolase	GO:0006954	inflammatory response	0.0202	0.0280	7	8	0	10	2	I
SJS/TEN	1DBJ	Fab′ Fragment of IgG	N/A	N/A	0.000439	0.00889	12	7	0	12	3	II
rhabdomyolysis	1B09	C-reactive protein	GO:0006953	acute-phase response	0.000135	0.00622	7	3	0	17	2	I
rhabdomyolysis	1DTL	Troponin T, Cardiac Muscle Isoforms	GO:0006937	regulation of muscle contraction	0.000182	0.00622	7	3	0	16	2	I
rhabdomyolysis	1F3M	PAK-1 protein kinase	GO:0008154	actin polymerization and/or depolymerization	0.00342	0.0177	4	0	4	20	1	I
rhabdomyolysis	2DDH	Acyl CoA oxidase 2	GO:0006629	lipid metabolic process	0.0235	0.0472	5	4	2	16	2	I
deafness	1BQS	Mucosal Addressin Cell Adhesion Molecule 1	GO:0007155	cell adhesion	5.93E-05	0.000597	10	2	3	21	2	I
deafness	1P4M	Riboflavin Kinase*	GO:0009231	riboflavin biosynthetic process	0.000322	0.00204	8	1	5	22	2	I
deafness	1IG3	Thiamin Pyrophosphokinase*	GO:0006772	thiamin metabolic process	0.000411	0.00236	9	2	4	20	2	I
deafness	1JJC	Phenylalanyl tRNA Synthetase	GO:0006412	translation	0.000434	0.00244	12	7	1	16	2	I
deafness	1JKL	Death-Associated Protein Kinase	GO:0006915	apoptosis	0.000920	0.00415	10	4	3	19	2	I
deafness	1BU5	Flavodoxin*	GO:0006810	transport	0.00636	0.0171	8	3	5	20	2	I
deafness	1EFR	Mitochondrial F1-ATPase*	GO:0006754	ATP biosynthetic process	0.00573	0.0206	7	5	2	18	1	I
cholestasis	1HN4	Prophospholipase A2	GO:0006633	fatty acid biosynthetic process	0.000347	0.0131	6	1	1	16	1	I
cholestasis	1HN5	Prophospholipase A3	GO:0050482	arachidonic acid secretion	0.000939	0.000939	7	1	3	16	2	I
cholestasis	1YTV	Vasopressin V1a receptor	GO:0006810	transport	0.000171	0.00531	11	4	1	15	3	I

See Table S5 for the full list of other class I and class II candidates.

*: Proteins contributing to energy metabolism specifically appeared in the protein set prioritized from deafness-oriented CPI (*p* = 0.0050, Chi-square test, two-tailed).

a The *q* value was calculated using the FDR correction algorithm [24].

### Identification of the candidate risk alleles of serious cutaneous reactions through insight into the CPI

**Figure 3:**
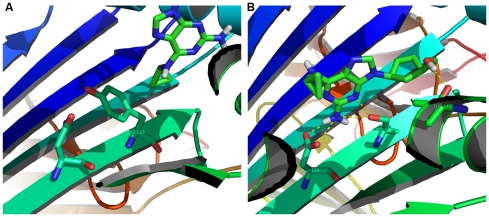
Models of abacavir's interactions with the binding site of HLA-B*5703 and HLA-B*5701. (*A*) The abacavir could not bind to the antigen presentation groove of MHC I (B*5703, PDB ID: 2BVP). No non-covalent interaction could be formed with N114 in the presence of Y116 due to the steric hindrance (Video S1). (*B*) The steric hindrance of the binding disappeared when Y116S was introduced. The binding score between abacavir and MHC I (B*5701) was much lower than with (B*5703) because of I) better compatibility of geometry shape to the binding pocket; II) hydrogen bonds (yellow dashed) were formed with three essential AA residues (D114, S116 and T143) within the antigen presentation groove (Video S2).

The CPI would not only tell which protein to mediate the SADR, but would also tell which allele of this protein would be more sensitive to the unexpected drug attack. To our knowledge, HLA-B*57 is the only reliable susceptibility gene of SADRs [32],[33] of which structures with both risk and non-risk alleles are available. We constructed an interactome including interaction strength among abacavir, allopurinol and four structures of risk and non-risk alleles of abacavir-induced hypersensitivity (Table 2). No specificity of allopurinol to any of the proteins was found. This result was in coordination with the fact that none of these alleles was the risk allele of allopurinol-induced hypersensitivity, In contrast, abacavir did not accommodate the binding site of B*5703, but appeared to have the high affinity with B*5701. The major difference between the two alleles lay in two polymorphisms (N114D, Y116S) from B*5703 to B*5701. When Y116S substitution appeared in B*5703, a better compatibility of geometry shape between drug and binding pocket as well as several hydrogen bonds were formed. As a result, abacavir molecule fixed deeply into the antigen presentation groove of HLA-B*57 (Figure 3, Video S2). Comparatively, the N114D seemed to be less important for the substitution of a nitrogen atom to an oxygen atom would neither affect the steric hindrance nor did the buildup of hydrogen bonds. Given the premise that direct binding of drug to HLA-B*57 protein mediates abacavir-induced hypersensitivity, we deduced that B*5701 tended to be the risk allele compared to B*5703. The discovery of the fact that abacavir interacts directly with the 114^th^ and 116^th^ residues of MHC I B*57 which mediates SJS is consistent with the genetic evidences [32]–[35]. This newly identified molecular mechanism has also been validated at the cell biology level [36]. In the presence of abacavir and MHC I (B*5703), the percentage of responding IFNg+ CD8+ T cells was only 1.34%, and the percentage remained unchanged when N114D mutation was introduced. However, this percentage suddenly rose to 28.4% when another mutation Y116S, which formed the risk allele B*5701, was introduced. The result fit the drug-MHC I direct binding model for that Y116S was essential to the binding of abacavir, and N114D tended to be less important.

**Table 2:** Interactome among abacavir, allopurinol and four HLA-B*57 molecules.

PDB ID	Allele	Dock Score^a^	Z-score	Is Risk Allele	Dock Score^b^	Z-score	Is Risk Allele
2BVO	B*5703	−33.73	0.460	No	−26.95	1.77	No
2BVQ	B*5703	−34.07	0.416	No	−28.56	0.141	No
2BVP	B*5703	−32.52	0.618	No	−26.88	1.84	No
2RFX	B*5701	−48.71	−1.49	Yes	−28.69	0.00565	No

a Docking score for drug abacavir.

b Docking score for drug allopurinol.

### Identification of candidate genes associated with cholestasis, rhabdomyolysis and deafness

A systematic insight into the CPI led to the identification of the SADR targets common to case drugs, and the risk alleles of them as well. We thus explored SADR targets for three other SADRs using the same methodology (Table 1). The relationships among drugs, SADR outcome and the corresponding sub-CPI were listed in Table S5. Vasopressin receptor was highlighted from cholestasis-related CPI. Being the native ligand, administration of vasopressin could result in a reduction in bile flow and then induce cholestasis [37]. Troponin T and PAK1 protein kinase were harvested as well. The former regulates muscle contraction while the latter, inhibited by most statin drugs in the rhabdomyolysis-oriented CPI, takes a vital part in the polymerization and depolymerization of actin. Energy dysfunction plays an important role in pathogenesis of hearing loss [38]. We found that, compared with other SADRs, deafness is significantly associated with proteins contributing to energy metabolism (see note of Table 1). The consistently higher than random recall rates of proteins known to be related to SADRs indicates that all these results could not have been achieved by chance.

### CPI profile of a drug reflects its interacting character towards multi-protein set

Network pharmacology [39] pointed out that many drug effects are mediated by the chemical-protein interactions of drugs towards multi-protein set. The SADR might also be triggered by the combination effect of drug-SADR target interactions. So we hypothesized that drugs causing the same SADR not only share the same SADR targets, but might also possess the similar binding strength profile towards multi-protein set. If this similarity can be detected, we may infer that the CPI not only represent the binding situation of a drug to a protein, but also reflect the interacting character of it towards multi-protein set. Here we utilized support vector machine (SVM) model to see whether drugs could be correctly classified as case or control drugs based on their binding profile vector against 845 proteins. If they could be, there was a similarity in high-dimensional space among case drugs or control drugs towards 845 protein set. The effectiveness of the classifier was measured by the binary classification accuracy using cross validation. For each SADR, categorical attributes of case or control drugs were labeled as “1” or “0”. Z-score of a drug towards 845 proteins was used as the attribute vector. The cross validation accuracy (CVA) varied from 85% to 91% among four SADRs (Table 3). To evaluate whether such CVAs were achieved by chance, we permutated the position of the case and control drugs randomly for 100 runs, and recalculated the mean CVAs. The mean CVAs of permutated data turned out to be much lower (Table 3). So we concluded that there were similarities among drugs with the same outcome of an SADR, which were found in the CPI profile of them towards multi-protein set.

**Table 3:** Comparison of the cross-validation accuracy (CVA) between the original dataset and the randomly permutated dataset.

SADR	Total Sample #	# of Case Drugs	# of Control Drugs	CVA (%)^a^	Mean CVA (%) of permutations
**SJS**	82	53	29	90.8	62.3
**cholestasis**	87	57	30	85.2	59.1
**deafness**	88	48	40	87.5	59.8
**rhabdomyolysis**	90	49	41	91.4	54.3

a Prediction models of the four SADRs shown here were validated by 5-fold cross validation.

Both case and control drug group contain the derivatives.

## Discussion

### The basic hypothesis of the CPI

The hypothesis that drugs with similar phenotypic effects tend to interact with same targets is similar to a recent study done by Campillos et al [40]. However, the target spaces and the aims of the two studies are different. The work of Campillos et al managed to construct new connections among drugs and known therapeutic targets, which are a small portion of protein spaces whose functional information is clearly identified. Our research tried to construct new connections among drugs and human proteins, which is a step into a larger protein space whose function needs further exploration. However, our methodology is hampered by the lack of the structurome information of the human proteins. The aim of the former research is to explore the off-targets. For our research, the major aim of finding the off-targets is to figure out the key interacting residues and the risk allele for each individual.

Unexpected drug-protein interaction is the vital step in pathogenesis of SADRs. Although drug response is a complex trait [1] mediated by multiple genes and their interactions, some well-known cases of polymorphism within a gene have pronounced effects on drug response. To our knowledge, all of these polymorphisms alter the pattern of direct chemical-protein interactions. Examples include the T790M mutation in the gefitinib binding pocket of EGFR [41]; the T164I mutation within the epinephrine binding pocket of β_2_-adrenergic receptor [42]; and the polymorphism within binding pocket of STI-571 to c-Abl [43]. Although multiple genes take part in immune response only the HLA genotypes are significantly associated with SJS [22],[32],[33],[44],[45]. Such a strong linkage suggests that a direct binding of SADR-causing drugs to MHC I may be the primary event. In the case of HLA-Cw*4, all sulfonamides bind to the antigen presentation groove. Several “wet” observations support this direct binding model. Firstly, the presentation of the sulfamethoxazole (SMX) parent drug displayed a direct, noncovalent binding fashion to the MHC–peptide complex [46]. Secondly, the antigen peptide within the MHC I groove does not appear to be essential, since the elution of the peptide did not affect the presentation of SMX [47], thus there might be competitive binding between the drugs and the peptide to the groove. Thirdly, von Greyerz *et al* [29] found that most T cell clones showed a “MHC-allele restricted drug-specific recognition” that was stimulated by the parent drug rather than its derivatives. Fourthly, Nassif [26] discovered that blister fluid T lymphocytes, which were derived from a patient suffering SMX-induced TEN, were cytotoxic only when SMX is present and the cells share HLA-Cw*4. This HLA allele-drug specific cytotoxicity was confirmed in another study [48], and can be abrogated with the change of a single residue in the groove. Finally, the S116Y within HLA-B*5701 was shown to hamper completely the presentation of abacavir [36], suggesting that the drug itself, or a metabolite, might be accommodated in the groove and the residue is essential for this binding. All these facts corroborate the direct drug-protein interaction model in which the strong MHC allele-drug specificity could be best explained by a steric complementarity together with other strong non-covalent interactions between the drug molecule and the antigen presentation groove. The groove contains a variable region where MHC I molecules coded by thousands of HLA alleles differ and the antigen peptide-MHC I recognition takes place. In our model, a similar drug-MHC I recognition occurs when the drug binds to its specific “port” of a particular MHC allele at the variant region. This specificity could also be found at the genetic level. For example, severe cutaneous adverse reactions are found to be triggered by allopurinol in the presence of B*5801 [44]; abacavir-induced skin reaction requires the parallel genotype of B*5701 [32]; and carbamazepin-induced SJS is linked to B*1502, but not to HLA-A*1101 [45]. All these markers have a pronounced predictive capability of SADRs, leading the U.S. FDA's recommendation for their implementation for personalized medicine. The growing body of evidence suggests that the direct chemical-protein binding may enable the identification of more promising markers for SADR genetics, especially for predicting the specific HLA alleles that may be responsible for other drug-induced hypersensitivity, and finally, for a better design of new drugs or the modification of existing drugs to prevent these unintended interactions.

### Availability and limitations of the CPI related techniques

Unexpected drug-protein interactions should be explored with any available technologies such as drug affinity pull-down [9] or compare the similarities of pocket shapes between known drug target and the off-target [49]. However, in a drug affinity pull-down experiment, it remains a challenging task to prioritize the candidate proteins and to explain the biological significance when hundreds of proteins are identified to be compatible to the drug. The CPI methodology of finding the common SADR target could enlighten the design of these “wet” experiments. For example, a pull-down through a mixture of resins immobilized by different case drugs might help to enrich the common targets, while the follow-up pull-down using resins immobilized by control drugs might exclude the false positives. Though DOCK could tell true bindings from the unidentified ones, no strict assessment had been put forward as to the resolving power of its scoring functions to evaluate the degree of the interaction strength of CPI. The lack of human protein structures is another problem. An ideal chemical-protein interactome would include the 3D structures of all human proteins whose structural flexibility can be effectively handled by more advanced docking programs so that the binding affinity can be better estimated. This would lead to a whole structurome-wide study with more unexpected interactions to be identified. Because of the biases in the structural and functional coverage in PDB and our pocket preparing criteria, the 845 proteins might not be representative, some of which were even redundant. The lack of randomization might introduce biases in the statistical model. Instead, preparing a reference protein set from other structural databases, e.g., choosing one representative structure for each SCOP super family [50], might improve the model. However, our pocket preparing criteria could guarantee all the proteins were targetable. Preserving the redundancy also enabled us to disclose more flexibility information of the protein cavities. Although this protein set needed improvement, with these structures, we were still able to i) highlight the true bindings from the unidentified bindings; ii) identify the susceptibility gene (HLA-Cw*4) for the SMX-induced TEN without any prior knowledge of the underlying mechanism; iii) identify the candidate risk allele of the susceptibility gene (HLA-B*5701) based on the direct drug-MHC I interaction model which had never been proposed in all drug-induced hypersensitivity models. In addition, many candidate SADR targets prioritized from the CPI tended to be linked to the known SADR mechanisms compared with the random selection. As “wet” techniques for building up the CPI is not mature, the *in silico* approach appears to be the only means feasible to apply the CPI at the prescreening level, considering the urgency of the global SADR problem. Though false positive candidate genes might also be proposed, they can be controlled in different data transformation steps. Firstly, if one ligand tends to give low docking scores, it is usually caused by the ligand factor which could be eliminated in the normalization steps. Secondly, if one target tends to give low docking scores, it could raise low score for both case and control drugs. So this target cannot achieve a low *p* value in Fisher's exact test, and cannot be highlighted in the CPI. Thirdly, the false positive given by *p* value judgment could be controlled by the FDR correction. Lastly, they can be eliminated through association studies of the SADR patients or the functional studies of the SADR mechanisms, just as the docking procedure for identifying drug candidates is always followed by the binding affinity experiments.

One limitation of our SVM classification model is the number of control samples in SJS/TEN group. We did not managed to find enough SJS- drugs to construct a sample set with case-control ratio at 1∶1. SJS seems to be particular that only a few drugs do not linked with it. We could not find enough independent validation set either due to our strict criteria of the sample selection. This is the first try of using CPI profile to predict ADR outcome of a drug, the prediction performance of the CPI thus needs further validation and the model needs to be optimized. However, the permutation result added some reliability to the classification model. Compared with the permutation result, the classification result showed there were similarities in the CPI profiles of the case drugs, which provided hint for the construction of the prediction model based on this methodology.

### Comparison with other techniques

Although case drug molecules in Fig. 2B shared some structure similarity, the high-dimension information provided by CPI extends beyond simple structural comparison. Take fluoxetine 4 and fluoxetine in Fig. 2A for example, only a simple change in the ionization state between the two molecules will give different CPI profiles, with the distance between the two molecules far on the clustering tree. The chemical-protein interactomic analysis might also be complementary to trascriptomics in toxicogenomics. The latter provides a rich description of cell status [51], whereas the CPI strategy seems to provide more direct and interpretable biological understandings. The primary interactions of a drug to proteins are the causes of biological events, whereas the trascriptome strategy only detects the resulting phenotypes. Knowing which proteins' function are disabled and which alleles tend to be disabled by the drugs are vital, for they might lay a direct solution to SADR at the source.

### Perspectives

Usually we could only access “wild-type” protein structures, and it is not only possible but also necessary to simulate genetic variability in 3D structures and thereby discover patient-specific off-targets so that we can predict one's SADR or drug effect from their “structypes”. However, we cannot construct a CPI that only uses the modeled “structypes”, since the type I error might accumulate significantly. So our strategy is to find proteins tend to be highlighted in a CPI containing “wild-type” structures, and then investigate whether the allele of the highlighted ones could constitute a risk allele.

The SADR genetics requires worldwide collaboration [52],[53]. At a time when samples are rare and the primary mechanisms are unclear, both the sample information and the hypothesized candidate genes should be shared by the community for prospective experimental validation. Thus, in anticipation of this global collaboration, we made the statistically significant candidate genes highlighted from CPI (Table S6) publicly available for SADR consortia's consideration, verification and the improvement of CPI methodology. We expect that the perfection of CPI will eventually benefit the public by understanding, minimizing and predicting the occurrence of SADRs.

## Methods

### Preparation of the protein pocket set and the ligand set

We selected targets from literature and from third-party targetable protein databases [14]–[17]. Every pocket had been examined manually and was screened according to the criteria pre-defined: I) In order to identify unexpected drug-protein interactions, the target space should not be confined to the narrow space of the targets for the marketed drugs, which are merely a small portion of all protein spaces. II) To utilize the structure space to the max, the species of the protein should not be confined to Homo Sapiens, and the homolog protein can be considered. Targets with greater than 30% in protein sequence similarity to the corresponding human protein at the bioactive site could be chosen. III) The PDB structure should contain the co-crystallized ligand to define the bioactive site and to indicate the protein is targetable, PDB entries whose ligand is at the surface of the protein are not acceptable. IV) The ligand embedded in the PDB structure should achieve certain rigidity and some specificity towards the target, e.g., compounds with a large portion of rotatable bonds were not acceptable. V) No missing residue should be around the bioactive site. Residues within 10 Å departed from the ligand were defined as the bioactive pocket of the protein, and balls with radius ranging from 1.1–1.4 Å were generated to fill in the pocket. Grid box was made 3–5 Å departed from the ‘cloud’ of the balls.

The SMILES information of the small molecules was retrieved from PubChem. The minimal energy conformations of chemicals were generated with CORINA. All structures of proteins and chemicals were prepared using Chimera [54] and PyMOL. All the above procedures were performed manually with a strict quality control.

### Construction of the test CPI

An intersection operation was performed between all drug targets in DrugBank and the proteins in our pocket set using PDB id or Uniprot id as the identifier. We confirmed that each of the proteins in intersection had at least two FDA-approved drugs bind directly to them with a clear pharmacology to insure that they were credible drug targets. The running of the DOCK program and the extraction of the results were controlled by Perl or shell scripts on a Ubuntu™ Linux cluster. The docking score was calculated as the sum of intramolecular and intermolecular energy. A docking score greater than zero was treated as a missing value. There were a total of 845 proteins with 891 pockets in our pocket set. When a protein has multiple pockets to bind with, only the lowest docking score was chosen as the reference score, so the docking score matrix was shrunk to 46×845 elements. Here X_ij_ represents the docking score of drug j to protein i. The Z-score is calculated as:
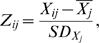
where
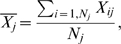


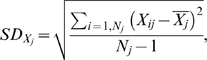
where N_j_ equals 845 minus the number of missing docking value of drug j to the protein set. So a Z-score matrix of 46×845 elements was generated. All direct bindings verified in literature or in the “description, indication, pharmacology and mechanism_of_action” fields of DrugBank database were defined as group 1, whereas other unidentified bindings were designated as group 2. The nonparametric Mann-Whitney test was performed on the Z-scores.

### Construction of a SADR oriented-CPI

All accessible AERS raw data from Jan 2004 to Mar 2008 were downloaded from FDA website and then deposited into a relational database (MySQL 5.1.22). In an SADR report, only the primary or the secondary suspected drugs were regarded as linked to the reported SADR. Drugs reported in the literature to have caused a SADR were further examined in AERS; only those reported in the AERS more than three times were considered as case drugs. The candidates for control drugs were collected on condition that they had never been co-cited with this SADR in the literature. They were classified into control group only if the report number was zero or less than 5% of its total reports when jointly used with the case drugs. The Z-score matrix was generated using the same pipeline as described above. However, for the SADR oriented-CPI, the Z-score matrix was further trimmed using the formula:
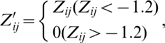
where Z_ij_ was the interaction strength between drug j and protein i. The Pearson correlation coefficient *r* between the two columns of binding affinity for drug j_1_ 
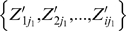
 and drug j_2_ 
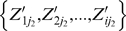
 was measured as:
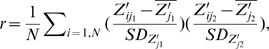
where N represents the number of Z′ value pairs of drug j_1_ and drug j_2_ with no missing value. 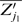 and 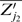 are the average Z′-score of drug j_1_ and j_2_, whereas 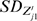 and 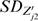 are standard derivation of Z′-score of the two drugs. The hierarchical clustering was then performed based on the *r* values between each pair of drugs. In the trimming procedure within each sub-CPI after clustering, only molecule with the lowest mean Z-score was chosen when multiple forms of a drug were clustered into the same sub-CPI. For protein i, *a*_i_, *b*_i_, *c*_i_, *d*_i_ values, representing the number of binding (*a*_i_ or *b*_i_) and non-binding (*c*_i_ or *d*_i_) by case drugs or control drugs respectively, were counted and the relative risk (RR) value was calculated as follows:
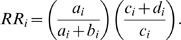
Protein targets with a RR value exceeding 1 were chosen for Fisher's exact test, as the expected values in any of the *a*_i_, *b*_i_, *c*_i_, *d*_i_ value sometimes is below 10. The *p* values were then corrected using fdrtool package [55] of R to control the false positives.

### Construction of SADR-oriented GCCN and enrichment analysis of GO terms

Although a more reliable network might be constructed using the STRING server [56], it is still uncertain that whether its physical and functional relationships can denote the true situation in SADR pathology. However, it could be more certain that a gene is involved in an SADR if it is cited specifically in this SADR related literature. So we only chose GCCN to organize the SADR related knowledge in a gene-oriented fashion. Four sets of PubMed entries were retrieved through the following four querying terms: Cholestasis; Deafness OR “hearing loss”; rhabdomyolysis OR myalgia OR myopathy OR myositis; rash OR Stevens-Johnson syndrome OR toxic epidermal necrolysis. The records were downloaded in XML format through the eSearch and eFetch APIs, and were deposited into a MySQL database. The index file of human genes to PubMed entries was downloaded from the Entrez Gene ftp site. A core gene of SADR “S” must meet one of the two criteria: the citation rate of this gene in this “S”-related corpus must exceed its citation rate in corpus under other topics; the number of citation must exceed four. A connection between two genes was established in GCCN if they were co-cited in more than two PubMed entries. Both core genes and extended genes of “S” were included in enrichment analysis of biological process (BP) GO terms using EASE [57]. The *p* value generated from Bonferroni correction was used as a measure in choosing the significant BP terms. The GO terms of each candidate proteins highlighted from CPI were assigned through querying the Gene Ontology Annotation database [58] with their UniProt ID as the identifier. Attributes of candidate class (I or II) were assigned manually depending on whether the candidate targets had shared the BP terms enriched from the GCCN.

### Construction of the binary classification model using the CPI profile

For each SADR, categorical attribute of a drug was labeled as “1” or “0” if it could (SADR+) or could not (SADR−) trigger this SADR. Z-scores were chosen as a measure of the interaction strength. Then for each protein, Z-scores were linearly scaled to the range of [−1, 1]. We chose a nonlinear RBF kernel function to build the model, because the relations between class labels and interactome attributes are nonlinear, and this kernel function,
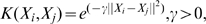
could map vectors onto a higher dimensional space nonlinearly. Here C and γ were two essential parameters for RBF function, but it was not known beforehand that which C and γ fitted best for our model. We performed the exhaustive searching for the best (C, γ) pair each time we performed the 5-fold cross-validation test.

## Supporting Information

Figure S1 ROC curve of the Z-score and the dock score in identifying the true bindings.
(0.03 MB DOC)

Figure S2 The lowest energy conformations of five sulfonamides' binding to the antigen presentation groove of MHC I (Cw*4). These five sulfonamides are piroxicam, sulfasalazine, tenoxicam, valdecoxib and tolbutamide. See Fig. 2C for the molecular structures of these drugs.
(0.44 MB DOC)

Figure S3 Molecular structures of the 17 control drugs which do not tend to interact with MHC I (Cw*4).
(0.16 MB DOC)

Table S1 Target set of the CPI.
(0.03 MB TXT)

Table S2 Mann-Whitney test of true positive bindings to the unidentified bindings on their Z-scores.
(0.03 MB DOC)

Table S3 The interaction strength among case-control drugs and HLA-Cw*4.
(0.06 MB DOC)

Table S4 T-test for equality of means on dock score and Z-score of HLA-Cw*4-oriented interactions between case drugs and control drugs.
(0.04 MB DOC)

Table S5 Drug molecules within each sub-CPIs after the trimming procedure.
(0.12 MB DOC)

Table S6 Candidate proteins for the four SADRs.
(0.35 MB DOC)

Video S1 Binding model of abacavir-MHC I B*5703.
(7.40 MB MOV)

Video S2 Binding model of abacavir-MHC I B*5701.
(7.43 MB MOV)
