# Supplementary material for: Harvesting Candidate Genes Responsible for Serious Adverse Drug Reactions from a Chemical-Protein Interactome
Source: PLoS Comput Biol. 2009 Jul 24;5(7):e1000441. doi: 10.1371/journal.pcbi.1000441 (PMC2704868; doi:10.1371/journal.pcbi.1000441)
Supplement: Figure S1 — ROC curve of the Z-score and the dock score in identifying the true bindings. (0.03 MB DOC) [file pcbi.1000441.s001.doc]

**Figure S1** ROC curve of the Z-score and the dock score in identifying the true bindings
