# Supplementary material for: Harvesting Candidate Genes Responsible for Serious Adverse Drug Reactions from a Chemical-Protein Interactome
Source: PLoS Comput Biol. 2009 Jul 24;5(7):e1000441. doi: 10.1371/journal.pcbi.1000441 (PMC2704868; doi:10.1371/journal.pcbi.1000441)
Supplement: Figure S2 — The lowest energy conformations of five sulfonamides' binding to the antigen presentation groove of MHC I (Cw*4). These five sulfonamides are piroxicam, sulfasalazine, tenoxicam, valdecoxib and tolbutamide. See Fig. 2C for the molecular structures of these drugs. (0.44 MB DOC) [file pcbi.1000441.s002.doc]

**Figure S2** The lowest energy conformations of five sulfonamides’ binding to the antigen presentation groove of MHC I (Cw*4)

These five sulfonamides are piroxicam, sulfasalazine, tenoxicam, valdecoxib and tolbutamide. See **Fig. 2*C*** for the molecular structures of these drugs.


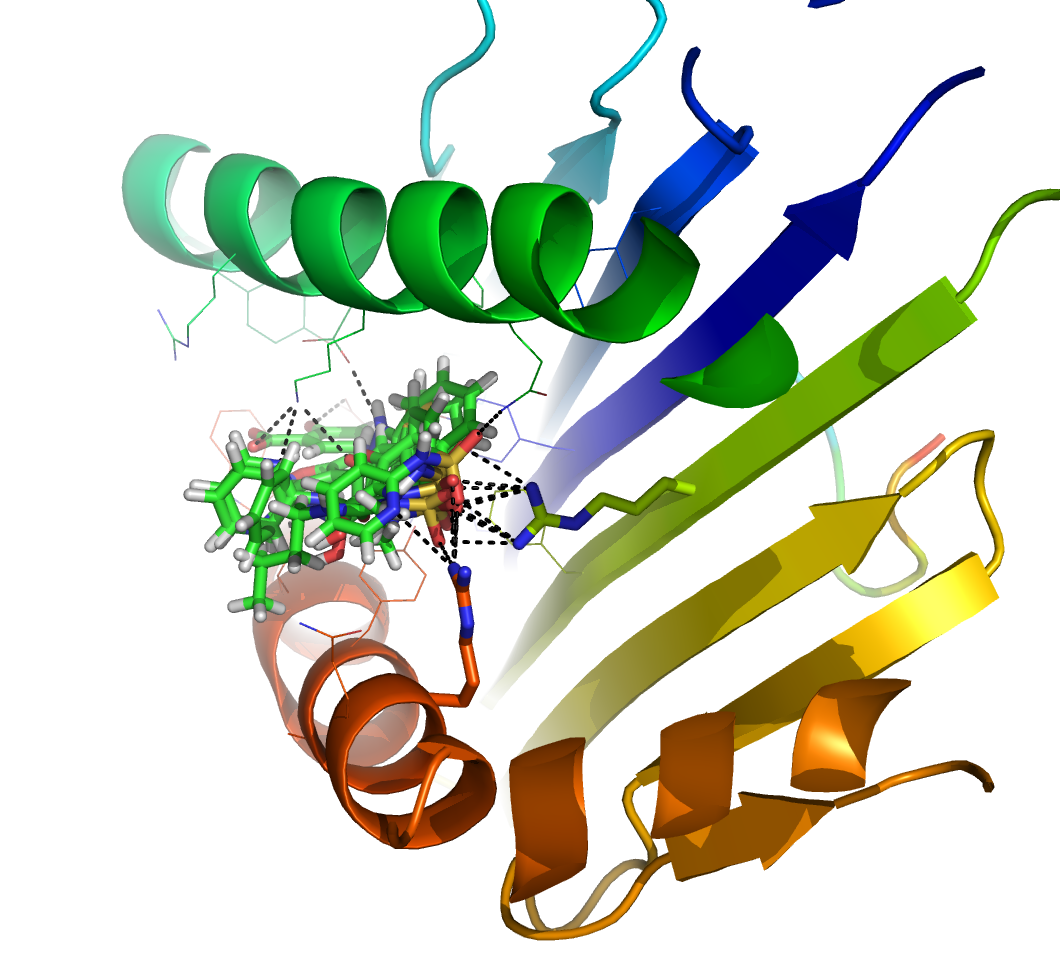


**R156**

**R97**
