# Supplementary figures and images for: Harvesting Candidate Genes Responsible for Serious Adverse Drug Reactions from a Chemical-Protein Interactome
Source: PLoS Comput Biol. 2009 Jul 24;5(7):e1000441. doi: 10.1371/journal.pcbi.1000441 (PMC2704868; doi:10.1371/journal.pcbi.1000441)

**Figure S3**
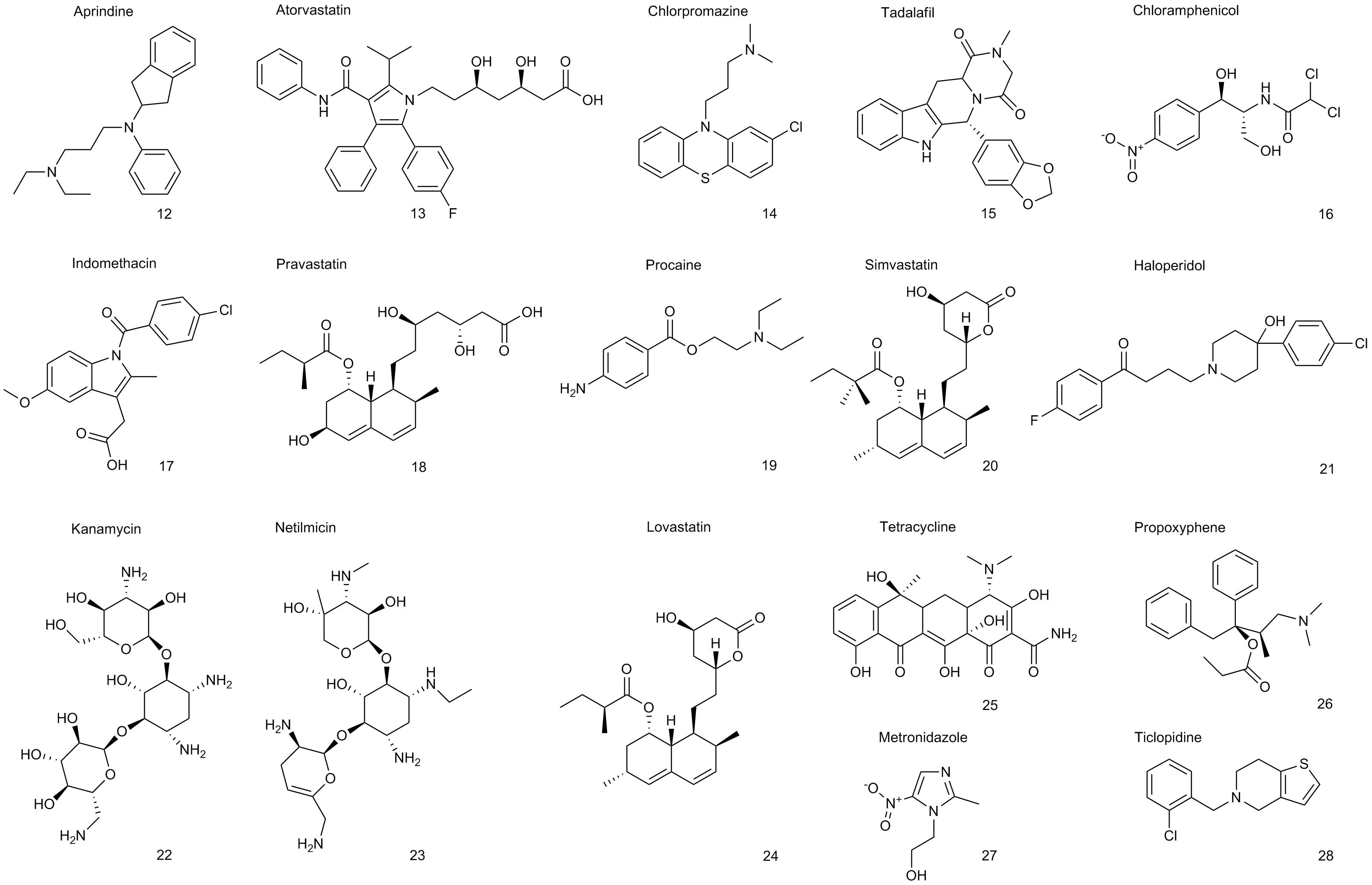
Molecular structures of the 17 control drugs which do not tend to interact with MHC I (Cw*4)

Supplement: Figure S3 — Molecular structures of the 17 control drugs which do not tend to interact with MHC I (Cw*4). (0.16 MB DOC) [file pcbi.1000441.s003.doc]
