# Supplementary material for: Harvesting Candidate Genes Responsible for Serious Adverse Drug Reactions from a Chemical-Protein Interactome
Source: PLoS Comput Biol. 2009 Jul 24;5(7):e1000441. doi: 10.1371/journal.pcbi.1000441 (PMC2704868; doi:10.1371/journal.pcbi.1000441)
Supplement: Table S2 — Mann-Whitney test of true positive bindings to the unidentified bindings on their Z-scores. (0.03 MB DOC) [file pcbi.1000441.s005.doc]

**Table S2** Mann-Whitney test of true positive bindings to the unidentified bindings on their Z-scores

|  | Group | N | Mean Rank | Sum of Ranks |
| --- | --- | --- | --- | --- |
| **Z-score** | 1^a^ | 53 | 139.33 | 7384.50 |
|  | 2 | 427 | 253.06 | 108055.50 |
|  | Total | 480 |  |  |

a Mann-Whitney U = 5953.500, *p* = 1.80E-8 (Asymp. Sig. 2-tailed).
