# Supplementary material for: Harvesting Candidate Genes Responsible for Serious Adverse Drug Reactions from a Chemical-Protein Interactome
Source: PLoS Comput Biol. 2009 Jul 24;5(7):e1000441. doi: 10.1371/journal.pcbi.1000441 (PMC2704868; doi:10.1371/journal.pcbi.1000441)
Supplement: Table S3 — The interaction strength among case-control drugs and HLA-Cw*4. (0.06 MB DOC) [file pcbi.1000441.s006.doc]

**Table S3** The interaction strength among case-control drugs and HLA-Cw*4

| **Drug Name** | **Drug Type** | **Dock Score** | **Z-score** |
| --- | --- | --- | --- |
| Celecoxib | case/sulfonamides | -50.40 | -1.410 |
| Piroxicam | case/sulfonamides | -54.67 | -1.484 |
| Sulfadoxine | case/sulfonamides | -49.05 | -1.243 |
| Sulfamethoxazole | case/sulfonamides | -50.76 | -1.613 |
| Sulfasalazine | case/sulfonamides | -62.75 | -1.608 |
| Tenoxicam | case/sulfonamides | -52.47 | -1.307 |
| Valdecoxib | case/sulfonamides | -47.03 | -1.251 |
| Bumetanide | case/sulfonamides | -59.35 | -1.340 |
| Tolbutamide | case/sulfonamides | -50.13 | -1.324 |
| Didanosine 3 | case/non-sulfonamides | -39.15 | -1.354 |
| ibuprofen 4 | case/non-sulfonamides | -30.20 | -1.220 |
| Diclofenac | case/non-sulfonamides | -35.27 | -0.668 |
| Valproate | case/non-sulfonamides | -27.67 | -1.096 |
| Aprindine | control | -18.85 | 0.567 |
| Atorvastatin | control | -33.94 | -0.849 |
| Chloramphenicol | control | -28.75 | -0.685 |
| Chlorpromazine | control | -21.50 | 0.644 |
| Haloperidol | control | -18.76 | 1.525 |
| Indomethacin | control | -35.33 | -0.670 |
| Kanamycin | control | -20.25 | 1.153 |
| Lovastatin | control | -21.92 | -1.071 |
| Metronidazole | control | -21.02 | -0.402 |
| Netilmicin | control | -25.86 | 0.484 |
| Pravastatin | control | -30.49 | -0.364 |
| Procaine | control | -23.51 | -0.491 |
| Propoxyphene | control | -14.29 | 0.847 |
| Simvastatin | control | -20.20 | -0.558 |
| Tadalafil | control | -28.72 | -1.136 |
| Tetracycline | control | -26.27 | 0.399 |
| Ticlopidine | control | -17.26 | 0.810 |

Drug names followed by the numbers represent the derivatives of this drug.
