# Supplementary material for: Harvesting Candidate Genes Responsible for Serious Adverse Drug Reactions from a Chemical-Protein Interactome
Source: PLoS Comput Biol. 2009 Jul 24;5(7):e1000441. doi: 10.1371/journal.pcbi.1000441 (PMC2704868; doi:10.1371/journal.pcbi.1000441)
Supplement: Table S4 — T-test for equality of means on dock score and Z-score of HLA-Cw*4-oriented interactions between case drugs and control drugs. (0.04 MB DOC) [file pcbi.1000441.s007.doc]

**Table S4** T-test for equality of means on dock score and Z-score of HLA-Cw*4-oriented interactions between case drugs and control drugs

|  | Drug Type | N | Mean | Std. Deviation |
| --- | --- | --- | --- | --- |
| Dock Score ^a^ | case | 9 | -52.9567 | 5.11976 |
|  | control | 17 | -23.9365 | 5.94016 |
| Z-score ^b^ | case | 9 | -1.3976 | .14173 |
|  | control | 17 | .0120 | .83217 |

a Dock scores in both case and control group accommodate to normal distribution. Equal variances were assumed (*F* = 0.566, *p* = 0.459). *t* = -12.394, *df* = 24, *p* = 6.38E-12.

b Z-scores in both case and control groups accommodate to normal distribution. Equal variances were assumed (*F* = 33.248, *p* = 6.07E-6). The corrected t-test was performed. *t* = -6.801, *df* = 17.695, *p* = 2.50E-6.
