# Supplementary material for: Harvesting Candidate Genes Responsible for Serious Adverse Drug Reactions from a Chemical-Protein Interactome
Source: PLoS Comput Biol. 2009 Jul 24;5(7):e1000441. doi: 10.1371/journal.pcbi.1000441 (PMC2704868; doi:10.1371/journal.pcbi.1000441)
Supplement: Table S5 — Drug molecules within each sub-CPIs after the trimming procedure. (0.12 MB DOC) [file pcbi.1000441.s008.doc]

**Table S5 Drug molecules within each sub-CPIs after the trimming procedure**

| **SADR** | **Drug Molecule** | **sub-CPI** |
| --- | --- | --- |
| cholestasis | Aspirin | 1 |
| cholestasis | Cytarabine 2 | 1 |
| cholestasis | Sulfasalazine | 1 |
| cholestasis | Captopril | 1 |
| cholestasis | Chlorothiazide | 1 |
| cholestasis | Tolbutamide | 1 |
| cholestasis | Sulfamethoxazole | 1 |
| cholestasis | Erythromycin | 2 |
| cholestasis | Aprindine | 2 |
| cholestasis | Cyproheptadine 2 | 2 |
| cholestasis | Haloperidol | 2 |
| cholestasis | Ticlopidine | 2 |
| cholestasis | Fluoxetine 3 | 2 |
| cholestasis | Amitriptyline | 2 |
| cholestasis | Chlorpromazine | 2 |
| cholestasis | Propoxyphene | 2 |
| cholestasis | Ampicillin | 2 |
| cholestasis | Amoxicillin | 2 |
| cholestasis | Trimethoprim | 3 |
| cholestasis | Fluoxetine | 3 |
| cholestasis | Phenobarbital | 3 |
| cholestasis | Carbamazepine | 3 |
| cholestasis | Azathioprine | 3 |
| cholestasis | Phenytoin | 3 |
| cholestasis | Phenobarbital 2 | 3 |
| cholestasis | Stavudine | 3 |
| cholestasis | Zidovudine | 3 |
| cholestasis | Nevirapine | 3 |
| cholestasis | Cytarabine | 3 |
| cholestasis | Didanosine | 3 |
| deafness | Indomethacin | 1 |
| deafness | Levitra | 1 |
| deafness | Vardenafil | 1 |
| deafness | Sildenafil 2 | 1 |
| deafness | Quinine | 1 |
| deafness | Furosemide | 1 |
| deafness | Bumetanide | 1 |
| deafness | Levofloxacin | 1 |
| deafness | Ciprofloxacin | 1 |
| deafness | Procaine 2 | 2 |
| deafness | Propranolol 2 | 2 |
| deafness | Chloroquine 2 | 2 |
| deafness | Tetracycline | 2 |
| deafness | Minocycline 2 | 2 |
| deafness | Doxycycline | 2 |
| deafness | Amikacin | 2 |
| deafness | Tobramycin | 2 |
| deafness | Kanamycin | 2 |
| deafness | Netilmicin | 2 |
| deafness | Gentamicin | 2 |
| deafness | Streptomycin | 2 |
| deafness | Erythromycin | 2 |
| deafness | Netilmicin 2 | 3 |
| deafness | Tobramycin 2 | 3 |
| deafness | Kanamycin 2 | 3 |
| deafness | Gentamicin 3 | 3 |
| deafness | Amikacin 2 | 3 |
| rhabdomyolysis | Atorvastatin | 1 |
| rhabdomyolysis | Bumetanide | 1 |
| rhabdomyolysis | Cerivastatin | 1 |
| rhabdomyolysis | Furosemide | 1 |
| rhabdomyolysis | Fluvastatin | 1 |
| rhabdomyolysis | Pravastatin 2 | 1 |
| rhabdomyolysis | Simvastatin 2 | 1 |
| rhabdomyolysis | Succinylcholine 2 | 1 |
| rhabdomyolysis | Methadone 4 | 2 |
| rhabdomyolysis | Chlorpromazine | 2 |
| rhabdomyolysis | Terbutaline 4 | 2 |
| rhabdomyolysis | Succinylcholine | 2 |
| rhabdomyolysis | Amphetamine 3 | 2 |
| rhabdomyolysis | Haloperidol | 2 |
| rhabdomyolysis | Risperidone 3 | 2 |
| rhabdomyolysis | Methadone | 3 |
| rhabdomyolysis | Terbutaline | 3 |
| rhabdomyolysis | Lovastatin | 3 |
| SJS/TEN | Diclofenac | 1 |
| SJS/TEN | Didanosine 3 | 1 |
| SJS/TEN | Sulfasalazine | 1 |
| SJS/TEN | Valdecoxib | 1 |
| SJS/TEN | Celecoxib | 1 |
| SJS/TEN | Valproate | 1 |
| SJS/TEN | Piroxicam | 1 |
| SJS/TEN | Tenoxicam | 1 |
| SJS/TEN | Sulfadoxine | 1 |
| SJS/TEN | Sulfamethoxazole | 1 |
| SJS/TEN | Ibuprofen 4 | 1 |
| SJS/TEN | Bumetanide | 1 |
| SJS/TEN | Tolbutamide | 1 |
| SJS/TEN | Erythromycin | 2 |
| SJS/TEN | Doxycycline | 2 |
| SJS/TEN | Minocycline | 2 |
| SJS/TEN | Fluoxetine 3 | 2 |
| SJS/TEN | Cephalexin | 2 |
| SJS/TEN | Amoxicillin | 2 |
| SJS/TEN | Ampicillin | 2 |
| SJS/TEN | Ciprofloxacin | 2 |
| SJS/TEN | Didanosine | 3 |
| SJS/TEN | Abacavir | 3 |
| SJS/TEN | Nevirapine | 3 |
| SJS/TEN | Fluoxetine | 3 |
| SJS/TEN | Flunitrazepam | 3 |
| SJS/TEN | Carbamazepine | 3 |
| SJS/TEN | Lamotrigine | 3 |
| SJS/TEN | Phenobarbital | 3 |
| SJS/TEN | Phenytoin | 3 |
| SJS/TEN | Fluconazole | 3 |
| SJS/TEN | Ibuprofen 3 | 3 |
| SJS/TEN | Ethambutol | 3 |
| SJS/TEN | Ethosuximide | 3 |

Drug names followed by the numbers represent the derivatives of this drug.
