# Supplementary material for: Harvesting Candidate Genes Responsible for Serious Adverse Drug Reactions from a Chemical-Protein Interactome
Source: PLoS Comput Biol. 2009 Jul 24;5(7):e1000441. doi: 10.1371/journal.pcbi.1000441 (PMC2704868; doi:10.1371/journal.pcbi.1000441)
Supplement: Table S6 — Candidate proteins for the four SADRs. (0.35 MB DOC) [file pcbi.1000441.s009.doc]

**Table S6** Candidate proteins for the four SADRs

| **SADR** | **PDB** | **Protein Name** | **GO ID** | **GO Term** | **p** | **q** | **a** | **b** | **c** | **d** | **sub-CPI** | **Type** |
| --- | --- | --- | --- | --- | --- | --- | --- | --- | --- | --- | --- | --- |
| SJS/TEN | 1QQD | Histocompatibility Leukocyte Antigen (Hla)-Cw*4(Heavy Chain) | GO:0006955 | immune response | 2.15E-05 | 2.98E-05 | 11 | 0 | 2 | 17 | 1 | I |
| SJS/TEN | 1QQD | Histocompatibility Leukocyte Antigen (Hla)-Cw*4(Heavy Chain) | GO:0019882 | antigen processing and presentation | 2.15E-05 | 2.98E-05 | 11 | 0 | 2 | 17 | 1 | I |
| SJS/TEN | 1OW3 | Rhoa.Gdp.Mgf3-in Complex With Rhogap | GO:0007165 | signal transduction | 2.30E-05 | 2.46E-04 | 12 | 1 | 1 | 16 | 1 | I |
| deafness | 1BQS | Protein (Mucosal Addressin Cell Adhesion Molecule-1) | GO:0007155 | cell adhesion | 5.93E-05 | 0.000597 | 10 | 2 | 3 | 21 | 2 | I |
| deafness | 1BQS | Protein (Mucosal Addressin Cell Adhesion Molecule-1) | GO:0006955 | immune response | 5.93E-05 | 0.000597 | 10 | 2 | 3 | 21 | 2 | I |
| deafness | 1O6L | Kt/Protein Kinase B (Pkb-pif Chimera) Ternary Complex With Amp-pnp And Gsk3 Peptide | GO:0043066 | negative regulation of apoptosis | 6.58E-05 | 0.000625 | 9 | 1 | 4 | 22 | 2 | I |
| rhabdomyolysis | 1B09 | Protein (C-reactive Protein) | GO:0006953 | acute-phase response | 0.000135 | 0.006216 | 7 | 3 | 0 | 17 | 2 | I |
| rhabdomyolysis | 1B09 | Protein (C-reactive Protein) | GO:0006954 | inflammatory response | 0.000135 | 0.006216 | 7 | 3 | 0 | 17 | 2 | I |
| cholestasis | 1YTV | Vasopressin V1a Receptor | GO:0006810 | transport | 0.000171 | 0.005307 | 11 | 4 | 1 | 15 | 3 | I |
| rhabdomyolysis | 1DTL | Cardiac Troponin C | GO:0006937 | regulation of muscle contraction | 0.000182 | 0.006216 | 7 | 3 | 0 | 16 | 2 | I |
| rhabdomyolysis | 1DTL | Cardiac Troponin C | GO:0032972 | regulation of muscle filament sliding speed | 0.000182 | 0.006216 | 7 | 3 | 0 | 16 | 2 | I |
| rhabdomyolysis | 1DTL | Cardiac Troponin C | GO:0055010 | ventricular cardiac muscle morphogenesis | 0.000182 | 0.006216 | 7 | 3 | 0 | 16 | 2 | I |
| deafness | 1P4M | Riboflavin Kinase | GO:0009231 | riboflavin biosynthetic process | 0.000322 | 0.002044 | 8 | 1 | 5 | 22 | 2 | I |
| cholestasis | 1HN4 | Prophospholipase A2 | GO:0006633 | fatty acid biosynthetic process | 0.000347 | 0.013057 | 6 | 1 | 1 | 16 | 1 | I |
| deafness | 1IG3 | Thiamin Pyrophosphokinase | GO:0006772 | thiamin metabolic process | 0.000411 | 0.002363 | 9 | 2 | 4 | 20 | 2 | I |
| deafness | 1JKL | Death-associated Protein Kinase | GO:0006915 | apoptosis | 0.00092 | 0.004151 | 10 | 4 | 3 | 19 | 2 | I |
| SJS/TEN | 2G5P | Dipeptidyl Peptidase 4 (CD26) | GO:0042110 | T cell activation | 9.39E-04 | 0.012422 | 7 | 3 | 1 | 16 | 2 | I |
| cholestasis | 1HN6 | Apical Membrane Antigen 1 | GO:0050778 | positive regulation of immune response | 0.000939 | 0.000939 | 7 | 1 | 3 | 16 | 3 | I |
| rhabdomyolysis | 1F3M | Serine/Threonine-protein Kinase Pak-alpha | GO:0006468 | protein amino acid phosphorylation | 0.003419 | 0.017741 | 4 | 0 | 4 | 20 | 1 | I |
| rhabdomyolysis | 1F3M | Serine/Threonine-protein Kinase Pak-alpha | GO:0008154 | actin polymerization and/or depolymerization | 0.003419 | 0.017741 | 4 | 0 | 4 | 20 | 1 | I |
| rhabdomyolysis | 1F3M | Serine/Threonine-protein Kinase Pak-alpha | GO:0006461 | protein complex assembly | 0.003419 | 0.017741 | 4 | 0 | 4 | 20 | 1 | I |
| rhabdomyolysis | 1B6C | Fk506-binding Protein | GO:0006468 | protein amino acid phosphorylation | 0.003419 | 0.017741 | 4 | 0 | 4 | 20 | 1 | I |
| deafness | 1EFR | Bovine Mitochondrial F1-atpase Complexed With The Peptide Antibiotic Efrapeptin | GO:0015992 | proton transport | 0.005726 | 0.020594 | 7 | 5 | 2 | 18 | 1 | I |
| deafness | 1EFR | Bovine Mitochondrial F1-atpase Complexed With The Peptide Antibiotic Efrapeptin | GO:0006754 | ATP biosynthetic process | 0.005726 | 0.020594 | 7 | 5 | 2 | 18 | 1 | I |
| deafness | 1EFR | Bovine Mitochondrial F1-atpase Complexed With The Peptide Antibiotic Efrapeptin | GO:0046034 | ATP metabolic process | 0.005726 | 0.020594 | 7 | 5 | 2 | 18 | 1 | I |
| deafness | 2AUH | Grb14 Bps Region In Complex With The Insulin Receptor Tyrosine Kinase | GO:0007169 | transmembrane receptor protein tyrosine kinase signaling pathway | 1.12E-02 | 0.027003 | 9 | 5 | 4 | 18 | 2 | I |
| rhabdomyolysis | 1XQZ | Proto-oncogene Serine/Threonine-protein Kinase Pim-1 | GO:0006468 | protein amino acid phosphorylation | 0.013804 | 0.032438 | 5 | 3 | 2 | 16 | 2 | I |
| SJS/TEN | 1HS6 | Leukotriene A-4 Hydrolase | GO:0006954 | inflammatory response | 0.020189 | 0.028005 | 7 | 8 | 0 | 10 | 2 | I |
| rhabdomyolysis | 2DDH | Acyl-coa Oxidase | GO:0006629 | lipid metabolic process | 0.023452 | 0.047229 | 5 | 4 | 2 | 16 | 2 | I |
| rhabdomyolysis | 2DDH | Acyl-coa Oxidase | GO:0006631 | fatty acid metabolic process | 0.023452 | 0.047229 | 5 | 4 | 2 | 16 | 2 | I |
| SJS/TEN | 1M4U | Bone Morphogenetic Protein-7 (Bmp-7) In Complex With The Secreted Antagonist Noggin | GO:0045596 | negative regulation of cell differentiation | 0.047555 | 0.219516 | 6 | 1 | 7 | 16 | 1 | I |
| SJS/TEN | 1XOS | Camp-specific 3',5'-cyclic Phosphodiesterase 4b | GO:0007165 | signal transduction | 7.81E-02 | 0.048051 | 5 | 4 | 3 | 14 | 2 | I |
| SJS/TEN | 1XOQ | Camp-specific 3',5'-cyclic Phosphodiesterase 4d | GO:0007165 | signal transduction | 0.089519 | 0.049686 | 6 | 6 | 2 | 12 | 2 | I |
| SJS/TEN | 2G63 | Dipeptidyl Peptidase 4 | GO:0042110 | T cell activation | 0.043255 | 0.039772 | 7 | 8 | 1 | 11 | 2 | I |
| SJS/TEN | 2OQV | Dipeptidyl Peptidase 4 (Dipeptidyl Peptidase Iv) (Dpp Iv) | GO:0042110 | T cell activation | 0.074303 | 0.047423 | 5 | 4 | 2 | 11 | 2 | I |
| SJS/TEN | 1DS6 | Rac-rhogdi Complex | N/A | N/A | 5.81E-06 | 2.00E-05 | 13 | 1 | 0 | 16 | 1 | II |
| SJS/TEN | 1A25 | Protein Kinase C (Beta) | N/A | N/A | 5.81E-06 | 2.00E-05 | 13 | 1 | 0 | 16 | 1 | II |
| rhabdomyolysis | 5ER2 | Endothiapepsin | N/A | N/A | 7.42E-06 | 0.000199 | 13 | 5 | 0 | 17 | 2 | II |
| cholestasis | 1GPN | Acetylcholinesterase | N/A | N/A | 1.00E-05 | 0.001863 | 11 | 2 | 1 | 17 | 3 | II |
| deafness | 5ER2 | Endothiapepsin | N/A | N/A | 1.28E-05 | 0.000226 | 13 | 6 | 0 | 17 | 2 | II |
| cholestasis | 5ER2 | Endothiapepsin | N/A | N/A | 1.28E-05 | 0.000226 | 13 | 6 | 0 | 17 | 2 | II |
| SJS/TEN | 1PIN | Peptidyl-prolyl Cis-trans Isomerase | N/A | N/A | 2.27E-05 | 2.98E-05 | 13 | 2 | 0 | 15 | 1 | II |
| SJS/TEN | 1MQ0 | Cytidine Deaminase | N/A | N/A | 2.27E-05 | 2.98E-05 | 13 | 2 | 0 | 15 | 1 | II |
| SJS/TEN | 1LIC | Adipocyte Lipid-binding Protein | N/A | N/A | 2.30E-05 | 0.000246 | 12 | 1 | 1 | 16 | 1 | II |
| SJS/TEN | 1BIF | 6-phosphofructo-2-kinase/ Fructose-2,6-bisphosphatase | N/A | N/A | 2.30E-05 | 0.000246 | 12 | 1 | 1 | 16 | 1 | II |
| deafness | 1KRA | Urease | N/A | N/A | 4.25E-05 | 0.000506 | 8 | 0 | 5 | 23 | 2 | II |
| cholestasis | 1XQZ | Proto-oncogene Serine/Threonine-protein Kinase Pim-1 | N/A | N/A | 5.00E-05 | 0.003871 | 11 | 4 | 0 | 14 | 2 | II |
| cholestasis | 1XQZ | Proto-oncogene Serine/Threonine-protein Kinase Pim-1 | N/A | N/A | 5.00E-05 | 0.003871 | 11 | 4 | 0 | 14 | 2 | II |
| deafness | 2BRO | Serine/Threonine-protein Kinase Chk1 | N/A | N/A | 5.93E-05 | 0.000597 | 10 | 2 | 3 | 21 | 2 | II |
| deafness | 1FYN | Phosphotransferase Fyn | N/A | N/A | 6.58E-05 | 0.000625 | 9 | 1 | 4 | 22 | 2 | II |
| cholestasis | 1MCS | Immunoglobulin Lambda Dimer Mcg (Light Chain) | N/A | N/A | 8.16E-05 | 0.004714 | 10 | 2 | 2 | 17 | 3 | II |
| deafness | 4ER2 | Endothiapepsin | N/A | N/A | 8.29E-05 | 0.000731 | 12 | 5 | 1 | 17 | 2 | II |
| SJS/TEN | 1H1B | Leukocyte Elastase | N/A | N/A | 8.52E-05 | 0.000246 | 10 | 0 | 3 | 17 | 1 | II |
| SJS/TEN | 1E1F | Beta-glucosidase | N/A | N/A | 8.83E-05 | 0.004159 | 12 | 5 | 0 | 14 | 3 | II |
| SJS/TEN | 1IX6 | Aspartate Aminotransferase | N/A | N/A | 0.000114 | 0.000246 | 11 | 1 | 2 | 16 | 1 | II |
| SJS/TEN | 1HKN | Heparin-binding Growth Factor 1 | N/A | N/A | 0.000114 | 0.000246 | 11 | 1 | 2 | 16 | 1 | II |
| SJS/TEN | 1GRE | Glutathione Reductase | N/A | N/A | 0.000114 | 0.000246 | 11 | 1 | 2 | 16 | 1 | II |
| SJS/TEN | 1UKE | Uridylmonophosphate/Cytidylmonophosphate Kinase | N/A | N/A | 0.00012 | 0.000246 | 12 | 2 | 1 | 15 | 1 | II |
| SJS/TEN | 1FT2 | Protein Farnesyltransferase | N/A | N/A | 0.000135 | 0.001273 | 13 | 4 | 0 | 13 | 1 | II |
| SJS/TEN | 1OOQ | Oxygen-insensitive Nad(P)H Nitroreductase | N/A | N/A | 0.000135 | 0.001273 | 13 | 4 | 0 | 13 | 1 | II |
| SJS/TEN | 2BZ1 | Gtp Cyclohydrolase Ii | N/A | N/A | 0.000135 | 0.001273 | 13 | 4 | 0 | 13 | 1 | II |
| cholestasis | 4ER2 | Endothiapepsin | N/A | N/A | 1.55E-04 | 0.008021 | 8 | 1 | 1 | 13 | 2 | II |
| deafness | 4ER1 | Endothiapepsin | N/A | N/A | 0.000155 | 0.008021 | 8 | 1 | 1 | 13 | 2 | II |
| cholestasis | 1E55 | Beta-glucosidase | N/A | N/A | 0.000171 | 0.005307 | 11 | 4 | 1 | 15 | 3 | II |
| cholestasis | 1DBJ | Igg1-kappa Db3 Fab (Light Chain) | N/A | N/A | 0.000171 | 0.005307 | 11 | 4 | 1 | 15 | 3 | II |
| deafness | 1JI4 | Neutrophil-activating Protein A | N/A | N/A | 0.000206 | 0.001511 | 7 | 0 | 6 | 23 | 2 | II |
| deafness | 1DHT | Estrogenic 17-beta Hydroxysteroid Dehydrogenase | N/A | N/A | 2.14E-04 | 0.023259 | 5 | 2 | 0 | 21 | 3 | II |
| cholestasis | 1ICN | Intestinal Fatty Acid Binding Protein | N/A | N/A | 2.73E-04 | 7.04E-03 | 9 | 1 | 3 | 15 | 3 | II |
| cholestasis | 1ACJ | Acetylcholinesterase | N/A | N/A | 0.000337 | 0.007838 | 12 | 3 | 0 | 8 | 3 | II |
| cholestasis | 4ER1 | Endothiapepsin | N/A | N/A | 0.000372 | 0.006331 | 7 | 4 | 0 | 16 | 2 | II |
| SJS/TEN | 1ICR | Oxygen-insensitive Nad(P)H Nitroreductase | N/A | N/A | 0.000409 | 0.001273 | 13 | 5 | 0 | 12 | 1 | II |
| SJS/TEN | 1BMQ | Protein (Interleukin-1 Beta Convertase) | N/A | N/A | 0.00042 | 0.001273 | 10 | 1 | 3 | 16 | 1 | II |
| SJS/TEN | 1QIP | Protein (Lactoylglutathione Lyase) | N/A | N/A | 0.00042 | 0.001273 | 10 | 1 | 3 | 16 | 1 | II |
| deafness | 1AM5 | Pepsin | N/A | N/A | 0.000434 | 0.002436 | 12 | 7 | 1 | 16 | 2 | II |
| SJS/TEN | 1DBJ | Igg1-kappa Db3 Fab (Light Chain) | N/A | N/A | 0.000439 | 0.008789 | 12 | 7 | 0 | 12 | 3 | II |
| SJS/TEN | 1DBK | Igg1-kappa Db3 Fab (Light Chain) | N/A | N/A | 0.000477 | 0.008994 | 8 | 1 | 4 | 18 | 3 | II |
| SJS/TEN | 1K86 | Caspase-7 | N/A | N/A | 0.000492 | 0.005483 | 12 | 3 | 1 | 14 | 1 | II |
| SJS/TEN | 2C6Q | Gmp Reductase 2 | N/A | N/A | 0.000492 | 0.005483 | 12 | 3 | 1 | 14 | 1 | II |
| SJS/TEN | 1HNA | Glutathione S-transferase | N/A | N/A | 0.000492 | 0.005483 | 12 | 3 | 1 | 14 | 1 | II |
| deafness | 1LT8 | Betaine-homocysteine Methyltransferase | N/A | N/A | 0.000627 | 0.00321 | 6 | 0 | 5 | 20 | 2 | II |
| SJS/TEN | 1IAY | 1-aminocyclopropane-1-carboxylate Synthase 2 | N/A | N/A | 0.000631 | 0.0094 | 8 | 0 | 3 | 14 | 1 | II |
| cholestasis | 2CGR | Igg2b-kappa Nc6.8 Fab (Light Chain) | N/A | N/A | 0.000635 | 0.011905 | 11 | 5 | 1 | 14 | 3 | II |
| cholestasis | 1GPK | Acetylcholinesterase | N/A | N/A | 0.000644 | 0.012003 | 10 | 1 | 2 | 10 | 3 | II |
| rhabdomyolysis | 3ER3 | Endothiapepsin | N/A | N/A | 0.000723 | 0.003544 | 13 | 10 | 0 | 13 | 2 | II |
| deafness | 1AH7 | Phospholipase C | N/A | N/A | 0.000723 | 0.003544 | 13 | 10 | 0 | 13 | 2 | II |
| rhabdomyolysis | 2HHA | Hypothetical Protein Dpp4 | N/A | N/A | 0.00081 | 0.008607 | 5 | 1 | 1 | 17 | 2 | II |
| rhabdomyolysis | 1CTR | Calmodulin | N/A | N/A | 0.000892 | 0.008855 | 7 | 5 | 0 | 15 | 2 | II |
| deafness | 1JKL | Death-associated Protein Kinase | N/A | N/A | 0.00092 | 0.004151 | 10 | 4 | 3 | 19 | 2 | II |
| deafness | 2YHX | Hexokinase B | N/A | N/A | 0.001126 | 0.012148 | 6 | 0 | 3 | 13 | 1 | II |
| SJS/TEN | 1JUE | Dihydroorotate Dehydrogenase A | N/A | N/A | 0.001277 | 0.0094 | 9 | 0 | 2 | 9 | 1 | II |
| deafness | 1I9C | Glutamate Mutase | N/A | N/A | 0.001282 | 0.033447 | 5 | 4 | 0 | 19 | 3 | II |
| deafness | 1JKL | Death-associated Protein Kinase | N/A | N/A | 0.001282 | 0.033447 | 5 | 4 | 0 | 19 | 3 | II |
| deafness | 1G1T | E-selectin | N/A | N/A | 0.001347 | 0.005365 | 8 | 2 | 5 | 21 | 2 | II |
| deafness | 1CTR | Calmodulin | N/A | N/A | 0.00135 | 0.005375 | 11 | 6 | 2 | 17 | 2 | II |
| deafness | 4DFR | Dihydrofolate Reductase | N/A | N/A | 0.001561 | 0.033978 | 5 | 4 | 0 | 18 | 3 | II |
| cholestasis | 1PQ3 | Arginase Ii, Mitochondrial Precursor | N/A | N/A | 0.001699 | 0.023918 | 10 | 5 | 1 | 14 | 2 | II |
| rhabdomyolysis | 1DMW | Phenylalanine Hydroxylase | N/A | N/A | 1.74E-03 | 0.010286 | 6 | 3 | 1 | 17 | 2 | II |
| SJS/TEN | 7AAT | Aspartate Aminotransferase | N/A | N/A | 0.001899 | 0.0094 | 10 | 2 | 3 | 15 | 1 | II |
| SJS/TEN | 1IA9 | Transient Receptor Potential-related Protein | N/A | N/A | 2.06E-03 | 1.23E-02 | 11 | 3 | 2 | 14 | 1 | II |
| SJS/TEN | 1C9C | Aspartate Aminotransferase | N/A | N/A | 2.06E-03 | 1.23E-02 | 11 | 3 | 2 | 14 | 1 | II |
| SJS/TEN | 2ANH | Alkaline Phosphatase | N/A | N/A | 2.06E-03 | 0.012333 | 11 | 3 | 2 | 14 | 1 | II |
| SJS/TEN | 1JK4 | Neurophysin 2 | N/A | N/A | 0.002226 | 0.012422 | 6 | 2 | 2 | 17 | 2 | II |
| rhabdomyolysis | 1J99 | Alcohol Sulfotransferase | N/A | N/A | 0.002341 | 0.016759 | 4 | 0 | 3 | 19 | 1 | II |
| SJS/TEN | 1ICP | 12-oxophytodienoate Reductase 1 | N/A | N/A | 0.00241 | 0.012333 | 10 | 2 | 3 | 14 | 1 | II |
| SJS/TEN | 1DGD | Dialkylglycine Decarboxylase | N/A | N/A | 0.002729 | 0.025402 | 7 | 0 | 6 | 17 | 1 | II |
| rhabdomyolysis | 1JTV | 17 Beta-hydroxysteroid Dehydrogenase Type 1 | N/A | N/A | 0.003047 | 0.01747 | 5 | 1 | 3 | 19 | 1 | II |
| deafness | 1LPM | Lipase | N/A | N/A | 0.003053 | 0.042325 | 3 | 0 | 2 | 23 | 3 | II |
| SJS/TEN | 2AEB | Arginase 1 | N/A | N/A | 0.003122 | 0.012422 | 8 | 7 | 0 | 12 | 2 | II |
| cholestasis | 2ACE | Acetylcholinesterase | N/A | N/A | 0.003329 | 0.040184 | 10 | 2 | 2 | 9 | 3 | II |
| deafness | 1LAY | Cytomegalovirus Protease | N/A | N/A | 3.41E-03 | 0.011057 | 5 | 0 | 8 | 23 | 2 | II |
| deafness | 1IH0 | Troponin C, Slow Skeletal And Cardiac Muscles | N/A | N/A | 0.003414 | 0.011057 | 5 | 0 | 8 | 23 | 2 | II |
| deafness | 2B3K | Methionine Aminopeptidase 1 | N/A | N/A | 0.003419 | 0.043524 | 4 | 2 | 1 | 21 | 3 | II |
| SJS/TEN | 1CBS | Cellular Retinoic Acid Binding Protein Type Ii | N/A | N/A | 0.003446 | 0.025402 | 7 | 0 | 6 | 16 | 1 | II |
| cholestasis | 1Q5M | Prostaglandin-e2 9-reductase | N/A | N/A | 0.003607 | 0.042011 | 11 | 4 | 1 | 9 | 3 | II |
| SJS/TEN | 2F4B | Peroxisome Proliferator-activated Receptor Gamma | N/A | N/A | 0.004077 | 0.025402 | 9 | 1 | 4 | 13 | 1 | II |
| cholestasis | 1CY1 | Dna Topoisomerase I | N/A | N/A | 0.004155 | 0.034167 | 6 | 1 | 4 | 16 | 2 | II |
| cholestasis | 2C0H | Mannan Endo-1,4-beta-mannosidase | N/A | N/A | 0.004167 | 0.034196 | 7 | 2 | 4 | 17 | 2 | II |
| deafness | 2ETR | Rho-associated Protein Kinase 1 | N/A | N/A | 0.00419 | 0.01273 | 9 | 4 | 4 | 18 | 2 | II |
| rhabdomyolysis | 1YMJ | Adp,Atp Carrier Protein, Heart Isoform T1 | N/A | N/A | 0.004236 | 0.020152 | 7 | 5 | 1 | 15 | 1 | II |
| rhabdomyolysis | 1DSY | Protein Kinase C, Alpha Type | N/A | N/A | 0.004652 | 0.018029 | 5 | 2 | 2 | 18 | 2 | II |
| SJS/TEN | 1ACM | Aspartate Carbamoyltransferase, Catalytic Chain | N/A | N/A | 0.004674 | 0.025402 | 8 | 1 | 5 | 16 | 1 | II |
| deafness | 1IGJ | Igg2a-kappa 26-10 Fab (Light Chain) | N/A | N/A | 0.004701 | 0.046518 | 5 | 6 | 0 | 17 | 3 | II |
| SJS/TEN | 2PK4 | Human Plasminogen Kringle 4 | N/A | N/A | 0.004785 | 0.013426 | 4 | 0 | 3 | 15 | 2 | II |
| deafness | 1OVM | Indole-3-pyruvate Decarboxylase | N/A | N/A | 0.004934 | 0.014148 | 6 | 1 | 7 | 22 | 2 | II |
| deafness | 1PPI | Alpha-amylase | N/A | N/A | 5.23E-03 | 0.014672 | 10 | 6 | 3 | 17 | 2 | II |
| deafness | 2ACS | Aldose Reductase | N/A | N/A | 0.005726 | 0.020594 | 7 | 5 | 2 | 18 | 1 | II |
| rhabdomyolysis | 1LBV | Fructose 1,6-bisphosphatase/Inositol Monophosphatase | N/A | N/A | 5.80E-03 | 2.02E-02 | 7 | 7 | 0 | 13 | 2 | II |
| SJS/TEN | 1C0N | Protein (Csdb Protein) | N/A | N/A | 0.005853 | 0.025402 | 9 | 2 | 4 | 15 | 1 | II |
| deafness | 1QD6 | Outer Membrane Phospholipase (Ompla) | N/A | N/A | 0.006356 | 0.017052 | 8 | 3 | 5 | 20 | 2 | II |
| SJS/TEN | 1JR1 | Inosine-5'-monophosphate Dehydrogenase 2 | N/A | N/A | 6.70E-03 | 0.025402 | 11 | 4 | 2 | 13 | 1 | II |
| SJS/TEN | 1JR1 | Inosine-5'-monophosphate Dehydrogenase 2 | N/A | N/A | 0.0067 | 0.025402 | 11 | 4 | 2 | 13 | 1 | II |
| SJS/TEN | 1K3Y | Glutathione S-transferase A1 | N/A | N/A | 0.0067 | 0.025402 | 11 | 4 | 2 | 13 | 1 | II |
| SJS/TEN | 1ZID | Enoyl-[Acyl-carrier-protein] Reductase | N/A | N/A | 0.0067 | 0.025402 | 11 | 4 | 2 | 13 | 1 | II |
| SJS/TEN | 1A2B | Transforming Protein Rhoa | N/A | N/A | 7.42E-03 | 7.68E-02 | 6 | 0 | 7 | 17 | 1 | II |
| SJS/TEN | 1ITU | Renal Dipeptidase | N/A | N/A | 8.47E-03 | 1.78E-02 | 7 | 5 | 1 | 14 | 2 | II |
| rhabdomyolysis | 1IMB | Inositol Monophosphatase | N/A | N/A | 0.008696 | 0.024097 | 6 | 5 | 1 | 15 | 2 | II |
| rhabdomyolysis | 1PPI | Alpha-amylase | N/A | N/A | 0.008696 | 0.024097 | 6 | 5 | 1 | 15 | 2 | II |
| rhabdomyolysis | 1BMQ | Protein (Interleukin-1 Beta Convertase) | N/A | N/A | 0.009466 | 0.033165 | 5 | 2 | 3 | 18 | 1 | II |
| rhabdomyolysis | 1D1T | Alcohol Dehydrogenase Class Iv Sigma Chain | N/A | N/A | 9.47E-03 | 0.033165 | 5 | 2 | 3 | 18 | 1 | II |
| rhabdomyolysis | 4TMN | Thermolysin | N/A | N/A | 0.009669 | 0.025342 | 5 | 4 | 1 | 16 | 2 | II |
| SJS/TEN | 1NHZ | Glucocorticoid Receptor | N/A | N/A | 0.010804 | 0.076815 | 8 | 1 | 5 | 13 | 1 | II |
| SJS/TEN | 1CRK | Creatine Kinase | N/A | N/A | 0.011392 | 0.020483 | 5 | 2 | 3 | 17 | 2 | II |
| deafness | 1G0Y | Interleukin-1 Receptor, Type I | N/A | N/A | 0.012138 | 0.02876 | 4 | 0 | 9 | 23 | 2 | II |
| SJS/TEN | 1ILH | Orphan Nuclear Receptor Pxr | N/A | N/A | 1.37E-02 | 0.130482 | 12 | 6 | 1 | 10 | 1 | II |
| cholestasis | 2AGT | Aldehyde Reductase | N/A | N/A | 0.013804 | 0.042902 | 5 | 3 | 2 | 16 | 1 | II |
| deafness | 1EED | Cyclohexyl Renin Inhibitor Pd125754 | N/A | N/A | 0.013986 | 0.031946 | 3 | 1 | 0 | 9 | 2 | II |
| deafness | 1JNK | C-jun N-terminal Kinase | N/A | N/A | 0.015017 | 0.036347 | 4 | 1 | 5 | 22 | 1 | II |
| deafness | 1BMM | Alpha-thrombin | N/A | N/A | 0.016078 | 0.035288 | 5 | 1 | 8 | 22 | 2 | II |
| rhabdomyolysis | 1E96 | The Rac/P67phox Complex | N/A | N/A | 0.017094 | 0.043265 | 3 | 0 | 5 | 20 | 1 | II |
| rhabdomyolysis | 1EVZ | Glycerol-3-phosphate Dehydrogenase | N/A | N/A | 1.71E-02 | 0.043265 | 3 | 0 | 5 | 20 | 1 | II |
| SJS/TEN | 1BID | Thymidylate Synthase | N/A | N/A | 0.018344 | 0.130482 | 9 | 3 | 4 | 14 | 1 | II |
| rhabdomyolysis | 1H82 | Polyamine Oxidase | N/A | N/A | 2.16E-02 | 4.47E-02 | 7 | 9 | 0 | 11 | 2 | II |
| cholestasis | 1DS6 | Rho | N/A | N/A | 0.022682 | 0.046753 | 7 | 9 | 0 | 10 | 1 | II |
| cholestasis | 1B74 | Glutamate racemase | N/A | N/A | 0.022682 | 0.046753 | 7 | 9 | 0 | 10 | 1 | II |
| SJS/TEN | 2A4Z | Phosphatidylinositol-4,5-bisphosphate 3-kinase Catalytic Subunit, Gamma Isoform | N/A | N/A | 0.023732 | 0.219516 | 5 | 0 | 8 | 16 | 1 | II |
| deafness | 1BMQ | Protein (Interleukin-1 Beta Convertase) | N/A | N/A | 0.038469 | 0.051006 | 4 | 2 | 5 | 21 | 1 | II |
| deafness | 1D2T | Acid Phosphatase | N/A | N/A | 0.038469 | 0.051006 | 4 | 2 | 5 | 21 | 1 | II |
| deafness | 1DHT | Estrogenic 17-beta Hydroxysteroid Dehydrogenase | N/A | N/A | 3.85E-02 | 0.051006 | 4 | 2 | 5 | 21 | 1 | II |
| SJS/TEN | 1AYL | Phosphoenolpyruvate Carboxykinase | N/A | N/A | 0.039454 | 0.219516 | 7 | 2 | 6 | 15 | 1 | II |
| SJS/TEN | 1A3G | Branched-chain Amino Acid Aminotransferase | N/A | N/A | 0.044127 | 0.040078 | 4 | 2 | 4 | 17 | 2 | II |
| SJS/TEN | 9AAT | Aspartate Aminotransferase | N/A | N/A | 0.047481 | 0.219516 | 9 | 4 | 4 | 13 | 1 | II |
| SJS/TEN | 1BGV | Glutamate Dehydrogenase | N/A | N/A | 0.048458 | 0.219516 | 10 | 5 | 3 | 12 | 1 | II |
| SJS/TEN | 1CVI | Prostatic Acid Phosphatase | N/A | N/A | 0.049587 | 0.219516 | 4 | 0 | 9 | 17 | 1 | II |
| SJS/TEN | 1GMN | Hepatocyte Growth Factor | N/A | N/A | 0.049587 | 0.219516 | 4 | 0 | 9 | 17 | 1 | II |
| SJS/TEN | 1KMQ | Transforming Protein Rhoa | N/A | N/A | 4.96E-02 | 0.219516 | 4 | 0 | 9 | 17 | 1 | II |
| SJS/TEN | 1AEC | Actinidin | N/A | N/A | 0.064615 | 0.045591 | 3 | 1 | 5 | 18 | 2 | II |
| SJS/TEN | 1IYH | Hematopoietic Prostagladin D Synthase | N/A | N/A | 0.07 | 0.046654 | 2 | 0 | 5 | 18 | 2 | II |
| SJS/TEN | 1MO7 | Sodium/Potassium-transporting Atpase Alpha-1 Chain | N/A | N/A | 7.98E-02 | 0.04831 | 2 | 0 | 6 | 19 | 2 | II |
